# The Effect of *Helicobacter pylori* Eradication on Lipid Levels: A Meta-Analysis

**DOI:** 10.3390/jcm10050904

**Published:** 2021-02-25

**Authors:** Jun Watanabe, Masato Hamasaki, Kazuhiko Kotani

**Affiliations:** Division of Community and Family Medicine, Jichi Medical University, 3311-1 Yakushiji, Shimotsuke-City, Tochigi 329-0498, Japan; m06105jw@jichi.ac.jp (J.W.); masatohamasaki@jichi.ac.jp (M.H.)

**Keywords:** cardiovascular disease, inflammation, *Helicobacter* infection, high-density lipoprotein, lipid metabolism, triglyceride

## Abstract

**Introduction:***Helicobacter pylori* (*H. pylori*) infection is positively associated with cardiovascular diseases, but the involvement of lipids in this association remains unclear. The present study reviewed the changes in circulating lipid levels following *H. pylori* eradication. **Methods:** A PubMed database was searched until December 2020 to identify randomized control trials (RCTs) and non-RCTs investigating the effect of *H. pylori* eradication on the lipid levels in inverse variance-weighted, random-effects meta-analyses. **Results:** A total of 24 studies (four RCTs and 20 non-RCTs) with 5270 participants were identified. The post-eradication levels were increased for high-density lipoprotein cholesterol (HDL-C; mean difference (MD) 2.28 mg/dL, 95% confidence interval (CI) 1.90 to 2.66) and triglyceride (TG; MD 3.22 mg/dL, 95% CI 1.13 to 5.31) compared with the pre-eradication levels. *H. pylori* eradication resulted in little to no difference in the low-density lipoprotein-cholesterol levels (MD −2.33 mg/dL, 95% CI −4.92 to 0.26). In the analyses of RCTs only, the findings for elevated HDL-C levels, but not TG, were robust. **Conclusions:** *H. pylori* eradication increases the HDL-C levels. Further studies are needed to elucidate the effects of lipid changes following *H. pylori* eradication on cardiovascular diseases.

## 1. Introduction

*Helicobacter pylori* (*H. pylori*) is a bacterium that causes chronic gastric inflammation [1,2]. A positive association of *H. pylori* infection with cardiovascular disease (CVD, e.g., myocardial infarction and stroke) has been recognized [2,3]. As the background theory, *H. pylori* infection is involved in the pathogenesis of atherosclerosis via activation of a local or systemic inflammatory host reaction and a subsequent induction of plaque progression and instability [2]. As abnormal lipid metabolisms contribute to the development of CVD [4,5,6,7,8,9,10], circulating lipids may be also involved in the relationship between *H. pylori* and CVD [11].

The existence of *H. pylori* infection was shown to be associated with a low level of high-density lipoprotein cholesterol (HDL-C), low-density lipoprotein (LDL-C), and total cholesterol (TC) or a high level of triglycerides (TG) in a recent meta-analysis of cross-sectional studies [11]. Nonetheless, the effect of *H. pylori* eradication on the lipid levels remains unclear. An earlier meta-analysis reported that *H. pylori* eradication did not alter the lipid levels [12]; however, that analysis included only three studies. Those three studies simply compared the lipid levels in the *H. pylori*-eradicated group with those in the *H. pylori*-negative group and did not compare the values before and after *H. pylori* eradication.

We investigated the changes in the levels of lipids after *H. pylori* eradication by reviewing meta-analyses of available published studies.

## 2. Methods

Candidate articles were searched via a PubMed search engine through 7 December 2020, using the following keywords: (“Cholesterol, HDL”[Mesh] OR “high-density lipoprotein cholesterol”[tiab] OR “Cholesterol, LDL”[Mesh] OR “low-density lipoprotein cholesterol”[tiab] OR “Triglycerides”[Mesh] OR “triglyceride”[tiab] OR “total cholesterol”[tiab] OR “Dyslipidemias”[Mesh] OR “Dyslipidemia”[tiab]) AND (“Helicobacter pylori”[Mesh] OR “Helicobacter pylori”[tiab]). Original articles that focused on *H. pylori* and lipid changes were included. No restrictions of age, gender, ethnicity, observation period, publication year to inclusion criteria were made. *H. pylori* infection was defined by positive results on either *H. pylori* IgG-antibody test, urea breath test, urease test, histology, culture, monoclonal stool antigen test, or a combination of these tests. Studies with data on pre- and post-lipid levels during *H. pylori* eradication and/or the change rates from baseline were included, and a sensitivity analysis was performed by using the changes from baseline. The exclusion criteria for the selection of articles were non-human and non-English articles. The reference lists of the extracted articles were hand-searched for further identification of additional studies.

The title and abstract of all candidate studies identified by the search were independently screened. The full texts of potentially relevant abstracts were assessed to determine whether or not the articles reported on the effect of *H. pylori* eradication on lipid changes. The study characteristics from the included studies were extracted, and a summary of tables was created in the quantitative synthesis. Meta-analyses were performed after excluding the articles that did not report the necessary outcomes and those with differing numbers of participants before and after *H. pylori* eradication.

The risk of bias was independently assessed using the Risk of Bias (RoB) 2 [13] in randomized controlled trials (RCTs) and the Newcastle-Ottawa Quality Rating Scale (NOS) [14] in non-randomized studies. In RoB 2, each of six domains (random sequence generation, allocation concealment, blinding of participants and personnel, blinding of outcome assessment, incomplete outcome data, and selective reporting) is classified into one of three categories (high risk, some concern, and low risk). The NOS assigns up to a maximum of 9 points to the highest-quality research according to three quality parameters (selection, comparability and outcome), with a higher score indicating a better quality. Any disagreement between the assessments of the two reviewers was resolved through discussion.

The mean difference (MD) with the 95% confidence interval (CI) of the mean change in HDL-C, TG, LDL-C, and total cholesterol (TC) after *H. pylori* eradication were calculated. The present meta-analysis was performed by comparing individual values before and after *H. pylori* eradication, regardless of the study design, as in the earlier studies [15,16]. The random-effects meta-analyses were performed in the Review Manager 5.4.1 software program (RevMan 2020) using the generic inverse variance method. The MD and standard deviation (SD) of continuous variables were integrated according to the method described in the Cochrane handbook [17]. The SD was substituted with the value from other studies when studies did not report the SD [17]. I^2^ statistics were used to assess statistical heterogeneity (I^2^ values of 0–40%: may not be important; 30–60%: may represent moderate heterogeneity; 50–90%: may represent substantial heterogeneity; 75–100%: may represent considerable heterogeneity) [17]. Sub-analyses were performed for the study design (RCTs, cohort studies, case–control studies, or before–after studies), follow-up duration (<1 or ≥1 year), and study location (Western or Asian countries). We performed sensitivity analyses to evaluate the robustness of our conclusions. This involved the exclusion of studies that did not describe the levels of HDL-C, TG, LDL-C, and TC before *H. pylori* eradication.

## 3. Results

**Figure 1:**
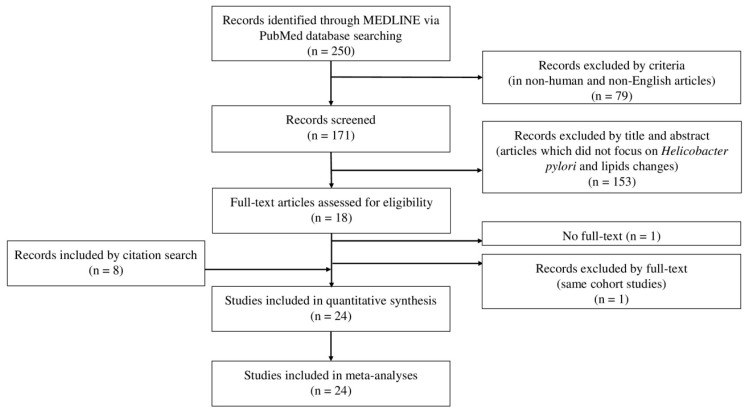
Flow chart of article selection.

Figure 1 shows the process for selecting articles that examined the effect of *H. pylori* eradication on lipid changes. A total of 250 records were initially screened. After screening, eight studies were identified that had been included during the electronic hand search, and two were excluded because one did not have the full text available [18] and the other involved the same cohort as another study already included in the review [19]. Ultimately, 24 studies that evaluated the effect of *H. pylori* eradication on lipid changes were included in our meta-analysis [20,21,22,23,24,25,26,27,28,29,30,31,32,33,34,35,36,37,38,39,40,41,42,43].

### 3.1. Mean Changes in Lipid Profiles after H. pylori Eradication

Table 1 summarizes the effect of *H. pylori* eradication on the lipid levels in 4 RCTs, 4 cohort studies, three case–control studies, and 13 before–after studies [20,21,22,23,24,25,26,27,28,29,30,31,32,33,34,35,36,37,38,39,40,41,42,43]. The median values for HDL-C, TG, LDL-C, and TC before *H. pylori* eradication were 52.1 (range: 30.1 to 64.8) mg/dL, 128 (range: 80.2 to 177.0) mg/dL, 122.2 (range: 101.8 to 146.0) mg/dL, and 195.0 (range: 158.0 to 212.1) mg/dL, respectively, in Appendix A: Table A1, Table A2, Table A3 and Table A4. The median age reported in the included studies was 48.4 (range: 29.1 to 56.7) years old. The treatment period ranged from 7 to 14 days. The median rate of *H. pylori* eradication was 84.0% (range: 54.7% to 100%).

The quality of the reviewed randomized and non-randomized studies was scored (Supplementary Materials Figures S1–S2). The overall risks of bias in the four RCTs were classified as low risk in one and some concern in three due to an unclear randomization process, missing outcome data, and no protocol. The quality assessment of the 20 non-RCTs included moderate scores ranging from 4 to 7. The main reason for the downgrade was an unclear medication status for dyslipidemia in 16 of the non-RCTs.

**Figure 2:**
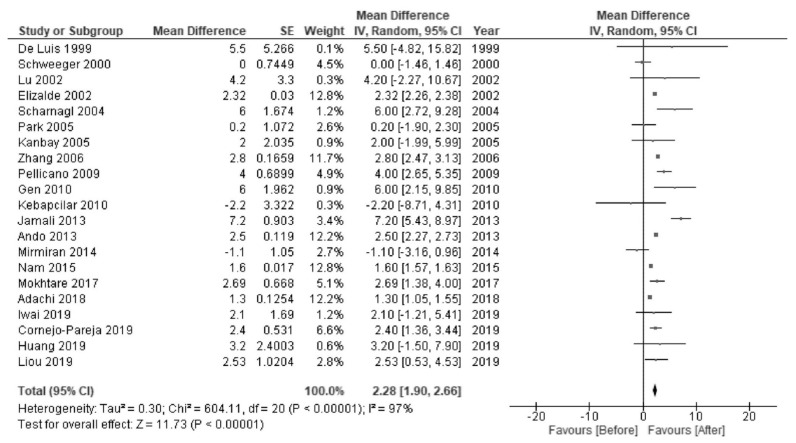
Forest plot of the mean changes in high-density lipoprotein cholesterol levels before and after *Helicobacter pylori* eradication.

**Figure 3:**
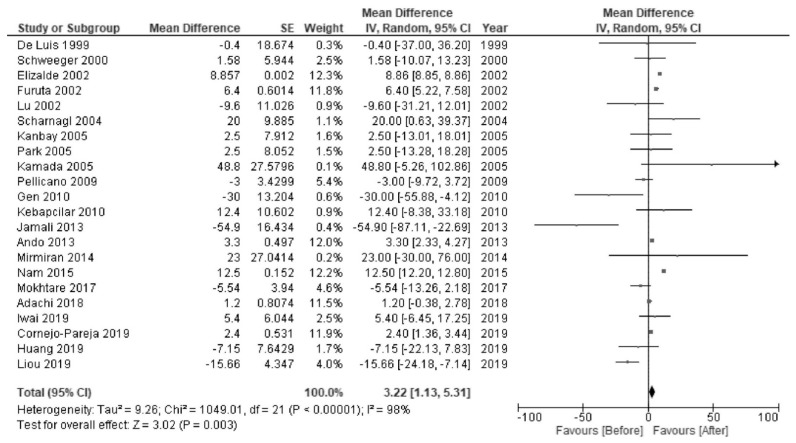
Forest plot of the mean changes in triglyceride levels before and after *Helicobacter pylori* eradication.

**Figure 4:**
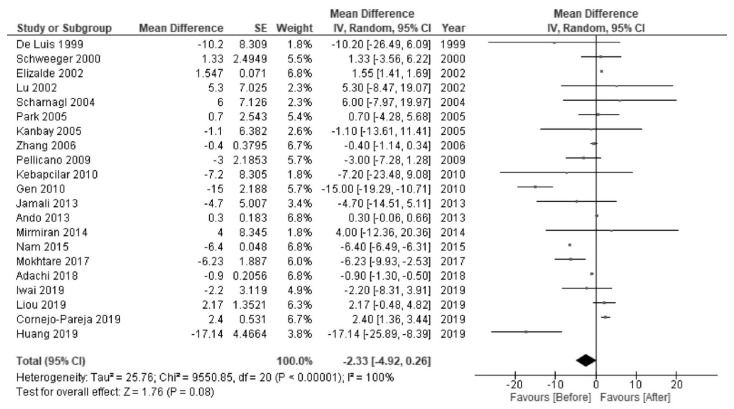
Forest plot of the mean changes in low-density lipoprotein cholesterol levels before and after *Helicobacter pylori* eradication.

**Figure 5:**
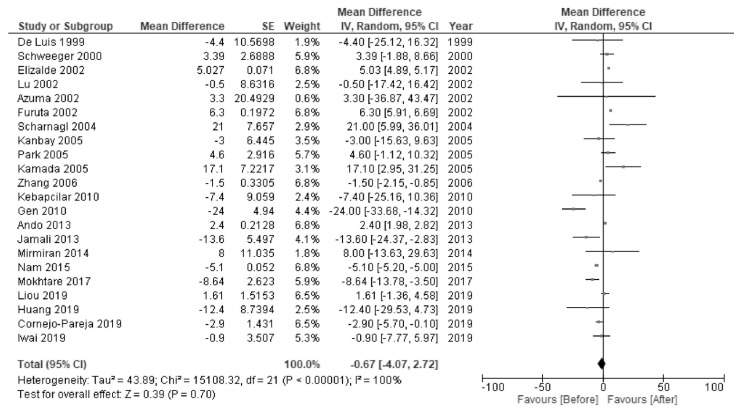
Forest plot of the mean changes in total cholesterol levels before and after *Helicobacter pylori* eradication.

*H. pylori* eradication was associated with increased levels of HDL-C (MD 2.28 mg/dL; 95% CI 1.90 to 2.66; I^2^ = 97%; Figure 2) and TG (MD 3.22 mg/dL; 95% CI 1.13 to 5.31; I^2^ = 98%; Figure 3). There were no significant changes in the levels of LDL-C (MD −2.33 mg/dL; 95% CI −4.92 to 0.26; I^2^ = 100%; Figure 4) or TC (MD −0.67 mg/dL; 95% CI −4.07 to 2.72; I^2^ = 100%; Figure 5).

### 3.2. Sub-Group Analyses

In RCTs, *H. pylori* eradication increased the HDL-C levels (MD 2.90 mg/dL; 95% CI 0.38 to 5.42; I^2^ = 92%; Supplementary Materials Figure S3). *H. pylori* eradication resulted in little to no difference in the levels of TG, LDL-C and TC (Supplementary Materials Figures S4–S6). In sub-group analyses of the follow-up period, *H. pylori* eradication was associated with increased HDL-C levels at follow-up periods of both <1 year (MD 2.35 mg/dL; 1.81 to 2.89; I^2^ = 80%) and ≥1 year (MD 2.26 mg/dL; 1.67 to 2.85; I^2^ = 91%) (Supplementary Materials Figure S7). The sub-group analyses of TG, LDL-C, and TC showed no significant differences (Supplementary Materials Figures S8–S10). In sub-group analyses by country (study location), *H. pylori* eradication increased the levels of HDL-C regardless of country: Western countries (MD 2.81 mg/dL; 95% CI 1.73 to 3.90; I^2^ = 83%) and Asian countries (MD 2.00 mg/dL; 95% CI 1.90 to 2.66; I^2^ = 93%) (Supplementary Figure S11). There were no significant effects of *H. pylori* eradication on the TG, LDL-C, or TC levels in Western or Asian countries (Supplementary Materials Figures S12–S14).

### 3.3. Sensitivity Analyses

The sensitivity analyses with the exclusion of studies that did not describe the levels of HDL-C, TG, LDL-C, or TC before *H. pylori* eradication were consistent with the primary findings that *H. pylori* eradication increased the HDL-C levels (Supplementary Materials Figures S15–S18).

## 4. Discussion

The present study meta-analyzed the changes in the lipid levels following *H. pylori* eradication. The post-eradication HDL-C levels were increased compared with pre-eradication. However, the post-eradication change in TG levels were unclear, as the results of sub-analyses were not consistent across study designs. The post-eradication LDL-C levels showed little to no change. Since *H. pylori* infection is positively associated with CVD [2,3], finding a relationship of HDL-C (as a protective factor of CVD) with *H. pylori* eradication, among the evaluated lipids, is of interest. Understanding the overall changes in lipids following *H. pylori* eradication may also help us ponder over the connection between bacteria and lipid metabolism.

The increase of 2.28 mg/dL of HDL-C by *H. pylori* eradication might be slight. Furthermore, recent evidence indicates that an increase of HDL-C with drug therapies may not always be beneficial of the prevention of CVD events [44], and in this line, not only HDL-C levels but also HDL functionals (e.g., anti-inflammatory/oxidant capacity, cholesterol efflux) may be crucial [44]. The clinical relevance of change of HDL-C by *H. pylori* eradication would be thus discussed. For instance, a 1 mg/dL increase of HDL-C is reported to result in a 3–5% reduction in CVD risk [45]. The linear inverse association between HDL-C and CVD events is also reported at least up to 90 mg/dL in HDL-C [46]. In lifestyle modifications against CVD, exercise and diet increased approximately 1 mg/dL in HDL-C [47] and smoking secession increased approximately 4 mg/dL [48]. Therefore, the changes in HDL-C levels by *H. pylori* eradication, as observed in the present review, is thought to have clinical relevance on CVD, while there have been no studies on CVD outcomes or HDL functions by HDL-C levels after *H. pylori* eradication.

*H. pylori* infection leads to systemic inflammation [49] and oxidative stress [50]. Inflammation and oxidative stress cause a reduction in HDL-C levels [51,52]. Cytokines induce the inflammatory molecule, serum amyloid A, which replaces a major HDL component (apolipoprotein A-I), thereby reducing the HDL-C levels [53]. In addition, inflammation and oxidative stress alter HDL-C-related enzymes; for example, lecithin-cholesterol acyltransferase, cholesterol ester transfer protein, and hepatic lipase levels are reduced while endothelial lipase levels are increased, which can reduce the HDL-C levels [54,55]. Abnormal cholesterol transport is another reason for the reduction in HDL-C levels, a phenomenon seen in *H. pylori* infection [39,40]. As another etiological mechanism, the microbiome may be involved [56]. *H. pylori* infection alters the gut microbiota [57], and eradication with antibiotic treatments has been shown to alter the microbiota and reduce *H. pylori*-induced inflammation [58]. Microbial diversity in the gut microbiota (a favorable phenomenon) can increase the HDL-C levels as it improves the suppression of lipid metabolism (with inflammation and oxidative stress) via bacteria-derived bile acids and short-chain fatty acids [59]. As above-mentioned, in recent evidence [44], not only HDL-C levels but also HDL functions affect the development of CVD. As the functions can be associated with inflammation, oxidative stress and gut microbiota [60,61], future work on HDL, in relation to biomolecules of inflammation/oxidative stress and gut environment, under *H. pylori* eradication is expected to explain comprehensively the results of the present review.

The existence of *H. pylori* infection was cross-sectionally associated with a high level of TG [11], and the present review found a slight but additional increase in TG levels following *H. pylori* eradication. *H. pylori* eradication increases the appetite [49,62] and improves nutrition [24,63,64], which can often promote obesity. Reductions in hepatic lipase and cholesterol ester transfer protein by the suppression of inflammation following *H. pylori* eradication or the reduction in the activity of lipoprotein lipase by obesity (insulin resistance) can lead to an increase of TG following *H. pylori* eradication [48,65,66]. However, according to the results of a sub-analysis of RCTs only, the TG levels were not increased following *H. pylori* eradication. This may be partly explained by the notion that physiological changes in TG levels are unstably affected by fluctuating factors, such as the diet and individual responses to the diet [67].

The levels of LDL-C and TC did not change markedly following *H. pylori* eradication, the reasons for which were unclear. First, interestingly, the degree of gastric mucosal atrophy is indicated to be associated with the LDL-C levels [37,39,68]. As advanced atrophy does not clearly change even after *H. pylori* eradication, this may partially explain the finding of no marked post-eradication changes in the levels of LDL-C or TC. Second, the type of antibiotics in the *H. pylori* eradication regimen might be considered because antibiotics, especially metronidazole, can reduce serum cholesterol, especially LDL-C levels [69]. In the present review, two studies using metronidazole reduced LDL-C [21,26]. Further studies focusing on the types of antibiotics in the *H. pylori* eradication regimen, especially metronidazole, are needed to see the changes in the LDL-C and TC levels after eradication.

In the present review, the heterogeneity of the analysis of the effects of *H. pylori* eradication on HDL-C appeared to be high. The results of a sub-analysis of RCTs showed high heterogeneity relative to non-RCTs. This heterogeneity might depend not only on the study design but also the follow-up period, as a sub-analysis based on the follow-up periods (short- and long-term after eradication) alleviated the heterogeneity. Antibacterial agents for *H. pylori* eradication are effective at eliminating inflammation in the short term, while improvements in gastric mucosal atrophy and microbial diversification are gradually seen in the long term [58,70,71].

Several limitations associated with the present study warrant mention. First, most studies were not controlled for covariates, such as underlying diseases related to lipid levels (e.g., liver cirrhosis, nephrotic syndrome) [72,73]; however, patients with such diseases might have been inadvertently excluded from the view of no indication of drugs for eradication. Second, the methods for detecting *H. pylori* infection (e.g., a urea breath test with a comparatively high accuracy among diagnostic methods [74] or a combination of tests) differed among studies. Third, the CVD outcomes following changes in lipid values after *H. pylori* eradication was not observed in all studies. Forth, we could not perform the subgroup analyses (i.e., participants’ sex, successful/unsuccessful eradication of *H. pylori*), which seemed to give more information, since the individual data were not obtained from the original studies or only limited data on such items were available in the reviewed studies. In the present review including 24 studies, there were only four non-RCTs with the data regarding successful/unsuccessful eradication therapy.

In conclusion, *H. pylori* eradication increased the HDL-C levels. The findings suggested that *H. pylori* eradication alters lipid profiles favorably for CVD. The results of more RCTs are required to derive more definitive conclusions.

## Figures and Tables

**Table 1:** Summary of the characteristics of the included studies.

Authors [ref no.]	Year	Design	Country	Subjects (*n*)	Age (Years)	Therapy Duration (Days)	Follow-Up (Months)	Eradication Rate (%)
De Luis [20]	1999	Before–after study	Spain	22	45.1	10	0.25	73.3
Schweeger [21]	2000	Before–after study	Austria	100	50.3	14	1	-
Azuma [22]	2002	Cohort study	Japan	241	42.9	7	12	79.8
Elizalde [23]	2002	Before–after study	Spain	368	47	7	0.25	79.0
Furuta [24]	2002	Cohort study	Japan	421	48	7	12	-
Lu [25]	2002	Before–after study	Taiwan	48	50.8	7	0.17	100
Scharnagl [26]	2004	Before–after study	Austria	87	52	7–14	1.00	-
Kamada [27]	2005	Cohort study	Japan	50	50.9	7	12	71.4
Kanbay [28]	2005	Before–after study	Turkey	57	49.9	14	0.17	100
Park [29]	2005	Case–control study	Korea	87	44.7	7	1.00	100
Zhang [30]	2006	Randomized control trial	China	136	51.1	14	48	-
Pellicano [31]	2009	Before–after study	Itary	496	59.7	-	<60	-
Gen [32]	2010	Before–after study	Tukey	47	32.8	14	6.00	54.7
Kebapcilar [33]	2010	Case–control study	Tukey	30	29.1	14	-	65.2
Ando [34]	2013	Before–after study	Japan	241	-	7	0.13	71.3
Jamali [35]	2013	Randomized control trial	Iran	48	43.8	14	0.46	96.0
Mirmiran [36]	2014	Randomized control trial	Iran	25	-	14	0.08	89.3
Nam [37]	2015	Cohort study	Korea	529	-	7	1–3	88.6
Mokhtare [38]	2017	Before–after study	Iran	91	41.2	14	0.25	62.6
Adachi [39]	2018	Cohort study	Japan	199	53.2	-	12	-
Iwai [40]	2019	Before–after study	Japan	163	56.7	14	1.41	84.0
Cornejo-Pareja [41]	2019	Case–control study	Spain	40	40	10	3	97.5
Hung [42]	2019	Cohort study	China	124	48.4	14	1	93.8
Liou [43]	2019	Randomized control trial	Taiwan	1620	53	10 or 14	12	87–95

*n*, number; ref no., reference number.

## Data Availability

Data have already been published in the academic literature.

## Supplementary Materials

The following are available online at https://www.mdpi.com/2077-0383/10/5/904/s1, Figure S1: Quality scores for the reviewed randomized studies, Figure S2: Quality scores for the reviewed non-randomized studies, Figure S3: Forest plot of the mean changes in high-density lipoprotein cholesterol levels before and after *Helicobacter pylori* eradication for each study design, Figure S4: Forest plot of the mean changes in triglyceride levels before and after *Helicobacter pylori* eradication for each study design, Figure S5: Forest plot of the mean changes in low-density lipoprotein cholesterol levels before and after *Helicobacter pylori* eradication for each study design, Figure S6: Forest plot of the mean changes in total cholesterol levels before and after *Helicobacter pylori* eradication for each study design, Figure S7: Forest plot of the mean changes in high-density lipoprotein cholesterol levels before and after *Helicobacter pylori* eradication during < 1- or ≥ 1-year follow-up, Figure S8: Forest plot of the mean changes in triglyceride levels before and after *Helicobacter pylori* eradication during < 1- or ≥ 1-year follow-up, Figure S9: Forest plot of the mean changes in low-density lipoprotein cholesterol levels before and after *Helicobacter pylori* eradication during < 1- or ≥ 1-year follow-up, Figure S10: Forest plot of the mean changes in total cholesterol levels before and after *Helicobacter pylori* eradication during < 1- or ≥ 1-year follow-up, Figure S11: Forest plot of the mean changes in high-density lipoprotein cholesterol levels before and after *Helicobacter pylori* eradication in Western countries or Asia, Figure S12: Forest plot of the mean changes in triglyceride levels before and after *Helicobacter pylori* eradication in Western countries or Asia, Figure S13: Forest plot of the mean changes in low-density lipoprotein cholesterol levels before and after *Helicobacter pylori* eradication in Western countries or Asia, Figure S14: Forest plot of the mean changes in total cholesterol levels before and after *Helicobacter pylori* eradication excluding studies which did not show the levels of HDL-C, TG, LDL-C and TC before *H. pylori* eradication, Figure S15: Forest plot of the mean changes in high-density lipoprotein cholesterol levels before and after *Helicobacter pylori* eradication excluding studies which did not show the levels of high-density lipoprotein cholesterol before *H. pylori* eradication, Figure S16: Forest plot of the mean changes in triglyceride levels before and after *Helicobacter pylori* eradication excluding studies which did not show the levels of triglycerides before *H. pylori* eradication, Figure S17: Forest plot of the mean changes in low-density lipoprotein cholesterol levels before and after Helicobacter pylori eradication excluding studies which did not show the levels of low-density lipoprotein cholesterol before H. pylori eradication.

## Appendix A

**Table A1:** Summary of levels of high-density lipoprotein cholesterol before and after eradication.

Authors (ref no.)	High-Density Lipoprotein Cholesterol Levels (mg/dL)		
	Before Eradication	After Eradication	Change Levels
De Luis [20]	59.7 ± 18.9	65.2 ± 15.9	5.5
Schweeger [21] *	45 ± 6 52 ± 5	45 ± 5 52 ± 5	0 0
Elizalde [23]	-	-	2.3
Lu [25]	49.7 ± 18.3	53.9 ± 13.7	4.2
Scharnagl [26]	36 ± 10	42 ± 12	6.0
Kanbay [28]	49.5 ± 10.3	51.5 ± 11.4	2.0
Park [29]	52.7 ± 13.7	-	0.2
Zhank [30]	56.8 ± 1.5	59.6 ± 1.2	2.8
Pellicano [31]	48	52	4.0
Gen [32]	37 ± 10	43 ± 9	6.0
Kebapcilar [33]	56.8 ± 13.5	54.6 ± 12.2	−2.2
Ando [34]	63.2 ± 1.33	65.7 ± 1.27	2.5
Jamali [35]	44.5 ± 5.7	51.7 ± 7.0	7.2
Mirmiran [36]	30.1 ± 4.0	29.0 ± 3.4	−1.1
Nam [37]	-	-	1.6
Mokhtare [38]	-	-	2.7
Adachi [39]	64.8 ± 1.2	66.1 ± 1.3	1.3
Iwai [40]	61.2 ± 14.7	63.3 ± 15.8	2.1
Cornejo-Pareja [41]	53.0 ± 2.0	55.4 ± 2.7	2.4
Haung [42]	33.6 ± 18.8	36.8 ± 19.0	3.2
Liou [43] **	54.2 ± 16.3 53.0 ± 16.0 52.1 ± 16.1	58.2 ± 18.8 55.8 ± 17.5 52.7 ± 16.3	4.0 3.1 0.6

ref no., reference number. * Smoker and non-smoker ** Triple therapy for 14 days, concomitant therapy for 10 days, and bismuth quadruple therapy.

**Table A2:** Summary of levels of triglycerides before and after eradication.

Authors [ref no.]	Triglycerides Levels (mg/dL)		
	Before Eradication	After Eradication	Change Levels
De Luis [20]	103.2 ± 67.1	102.8 ± 56.3	−0.4
Schweeger [21] *	171 ± 47 124 ± 36	169 ± 51 128 ± 37	−2.0 4.0
Elizalde [23]	-	-	8.9
Furuta [24]	120.0 ± 10.0	126.4 ± 7.23	6.4
Lu [25]	110.5 ± 58.6	100.9 ± 49.0	−9.6
Scharnagl [26]	128 ± 55	148 ± 74	20.0
Kamada [27]	125.3 ± 67.4	174.1 ± 183	48.8
Kanbay [28]	127.9 ± 63.7	129.6 ± 49.3	2.5
Park [29]	177.0 ± 192.9	-	2.5
Pellicano [31]	132	129	−3.0
Gen [32]	159 ± 63	129 ± 65	−30.0
Kebapcilar [33]	80.2 ± 30.2	92.6 ± 49.6	12.4
Ando [34]	109.3 ± 4.1	112.6 ± 6.6	3.3
Jamali [35]	174.8 ± 103.2	119.9 ± 48.1	−54.9
Mirmiran [36]	131 ± 80	154 ± 109	23.0
Nam [37]	-	-	12.5
Mokhtare [38]	-	-	−5.5
Adachi [39]	110.1 ± 7.8	111.3 ± 8.3	1.2
Iwai [40]	98.1 ± 50.9	103.5 ± 58.0	5.4
Cornejo-Pareja [41]	97.2 ± 6.3	93.5 ± 5.9	2.4
Huang [42]	157.5 ± 61.3	150.3 ± 59.1	−7.2
Liou [43] **	128.1 ± 94.8 139.1 ± 106.0 141.2 ± 97.6	119.0 ± 75.8 115.4 ± 63.0 127.0 ± 81.6	−9.1 −23.7 −14.2

ref no., reference number. * Smoker and non-smoker ** Triple therapy for 14 days, concomitant therapy for 10 days, and bismuth quadruple therapy.

**Table A3:** Summary of levels of low-density lipoprotein cholesterol before and after eradication.

Authors [ref no.]	Low-Density Lipoprotein Cholesterol Levels (mg/dL)		
	Before Eradication	After Eradication	Change Levels
De Luis [20]	111.9 ± 28.2	101.7 ± 26.9	−10.2
Schweeger [21] *	129 ± 17 114 ± 17	128 ± 18 118 ± 17	−1.0 4.0
Elizalde [23]	-	-	1.5
Lu [25]	135.1 ± 37.6	140.4 ± 30.9	5.3
Scharnagl [26]	146 ± 47	157 ± 47	6.0
Kanbay [28]	125.4 ± 32.8	124.3 ± 35.3	−1.1
Park [29]	123.6 ± 27.1	-	0.7
Zhang [30]	115.6 ± 3.1	115.2 ± 3.1	−0.4
Pellicano [31]	140	137	−3.0
Gen [32]	119 ± 12	104 ± 9	−15.0
Kebapcilar [33]	111.7 ± 33.1	104.5 ± 31.2	−7.2
Ando [34]	124.7 ± 2.0	125.0 ± 2.0	0.3
Jamali [35]	101.8 ± 25.7	91.5 ± 19.3	−4.7
Mirmiran [36]	104 ± 29	108 ± 30	4.0
Nam [37]	-	-	−6.4
Mokhtare [38]	-	-	−6.2
Adachi [39]	131.1 ± 2.1	130.2 ± 2.0	−0.9
Iwai [40]	121.2 ± 28.7	119.0 ± 27.6	−2.2
Cornejo-Pareja [41]	121.5 ± 5.7	118.0 ± 5.2	2.4
Huang [42]	128.4 ± 39.4	111.3 ± 30.3	−17.1
Liou [43] **	122.2 ± 33.3 121.0 ± 31.0 122.3 ± 33.9	123.9 ± 34.0 124.1 ± 33.5 123.9 ± 34.3	1.7 3.1 1.6

ref no., reference number. * Smoker and non-smoker ** Triple therapy for 14 days, concomitant therapy for 10 days, and bismuth quadruple therapy.

**Table A4:** Summary of levels of total cholesterol before and after eradication.

Authors (ref no.)	Total Cholesterol Levels (mg/dL)		
	Before Eradication	After Eradication	Change Levels
De Luis [20]	191.2 ± 35.7	186.8 ± 34.4	−4.4
Schweeger [21] *	215 ± 25 196 ± 17	216 ± 19 201 ± 16	1.0 5.0
Azuma [22]	200.8 ± 31.6	204.1 ± 36.8	3.3
Elizalde [23]	-	-	5.0
Furuta [24]	191.1 ± 3.1	197.4 ± 2.6	
Lu [25]	209.2 ± 44.0	208.7 ± 40.5	−0.5
Scharnagl [26]	208 ± 50	229 ± 51	21.0
Kamada [27]	204.1 ± 33.2	221.2 ± 38.8	17.1
Kanbay [28]	208 ± 33.8	205 ± 35	−3.0
Park [29]	212.1 ± 38.7	-	4.6
Zhang [30]	190.6 ± 2.7	189.1 ± 2.7	−1.5
Gen [32]	212 ± 28	188 ± 19	−24.0
Kebapcilar [33]	180.2 ± 31.3	172.8 ± 38.5	−7.4
Ando [34]	209.9 ± 2.27	212.3 ± 2.4	2.4
Jamali [35]	180.8 ± 31.9	167.2 ± 20.8	−13.6
Mirmiran [36]	158 ± 38	166 ± 40	8.0
Nam [37]	-	-	−5.1
Mokhtare [38]	-	-	−8.6
Iwai [40]	206.0 ± 32.5	205.1 ± 30.8	−0.9
Cornejo-Pareja [41]	194.2 ± 6.5	191.3 ± 6.3	−2.9
Huang [42]	182.7 ± 70.2	170.3 ± 67.4	−12.4
Liou [43] **	194.8 ± 38.4 194.0 ± 35.0 195.0 ± 37.0	197.0 ± 36.6 196.7 ± 37.5 196.0 ± 38.9	1.0 2.7 1.0

ref no., reference number. * Smoker and non-smoker ** Triple therapy for 14 days, concomitant therapy for 10 days, and bismuth quadruple therapy.
