# Supplementary material for: The Effect of Helicobacter pylori Eradication on Lipid Levels: A Meta-Analysis
Source: J Clin Med. 2021 Feb 25;10(5):904. doi: 10.3390/jcm10050904 (PMC7956592; doi:10.3390/jcm10050904)
Supplement: Supplementary file 1 [file jcm-10-00904-s001.pdf]

Journal of
Clinical Medicine

MDPI

Authors [reference number]	Risk of bias 2 tool assessment					
	Bias arising from the randomization process	Bias due to deviations from intended interventions	Bias due to missing outcome data	Bias in measurement of the outcome	Bias in selection of the reported result	Overall risk of bias
Zhang [30]	+	+	?	?	-	-
Jamali [35]	+	+	+	?	-	-
Mirmiran [36]	+	+	+	+	-	-
Liou [43]	+	+	+	?	-	-

\+ Low risk ? Some concerns - High risk

Supplementary Figure S1. Quality scores for the reviewed randomized studies

	The Newcastle-Ottawa Quality Assessment Scale							
	Selection			Comparability		Outcome		Total
Authors [ref no.]	Representatives of the exposed cohort/ adequate case difinition	Selection of the non-exposed cohort/ representative of cases	Ascertainment of exposure/ selection of controls	Demonstration that outcome of interest was not present at start of study/ definition of controls	Comparability on the basis of design or analysis	Assessment of outcome/ exposure	Was follow-up long enough for outcomes to occur	Adequacy of follow-up of cohorts	Score
De Luis [20]	★	-	★	-	-	★	-	★	4
Schweeger [21]	★	★	★	★	★	★	-	★	7
Azuma [22]	★	★	★	-	-	★	★	★	6
Elizalde [23]	★	★	★	-	-	★	-	★	5
Furuta [24]	★	★	★	-	-	★	★	★	6
Lu [25]	★	★	★	-	-	★	-	★	5
Scharnagl [26]	★	★	★	-	-	★	-	★	5
Kamada [27]	★	-	★	-	-	★	★	★	5
Kanbay [28]	★	★	★	★	★	★	-	★	7
Park [29]	★	★	★	-	-	★	-	★	5
Pellicano [31]	★	★	★	-	-	★	★	★	6
Gen [32]	★	-	★	★	★	★	★	★	7
Kebapcilar [33]	★	★	★	★	★	★	-	-	6
Ando [34]	★	★	★	-	-	★	-	-	4
Nam [37]	★	★	★	-	-	★	-	★	5
Mokhtare [38]	★	★	★	-	-	★	-	★	5
Adach [39]	★	★	★	-	-	★	★	-	5
Iwai [40]	★	★	★	-	-	★	-	★	5
Cornejo-Pareja [41]	★	★	★	-	-	★	-	★	5
Hung [42]	★	-	★	-	-	★	★	★	5

★ was awarded when the respective information was available.

Supplementary Figure S2. Quality scores for the reviewed non-randomized studies

Journal of
Clinical Medicine

Study or Subgroup	Mean Difference	SE	Weight	Mean Difference IV, Random, 95% CI	Year
**1.1.1 Randomized control trial**					
Zhang 2006	2.8	0.1659	11.7%	2.80 [2.47, 3.13]	2006
Jamali 2013	7.2	0.903	3.4%	7.20 [5.43, 8.97]	2013
Mirmiran 2014	-1.1	1.05	2.7%	-1.10 [-3.16, 0.96]	2014
Liou 2019	2.53	1.0204	2.8%	2.53 [0.53, 4.53]	2019
**Subtotal (95% CI)**			**20.7%**	**2.90 [0.38, 5.42]**	
Heterogeneity: Tau² = 5.89; Chi² = 37.54, df = 3 (P < 0.00001); I² = 92%					
Test for overall effect: Z = 2.25 (P = 0.02)					
**1.1.2 Cohort study**					
Nam 2015	1.6	0.017	12.8%	1.60 [1.57, 1.63]	2015
Adachi 2018	1.3	0.1254	12.2%	1.30 [1.05, 1.55]	2018
Huang 2019	3.2	2.4003	0.6%	3.20 [-1.50, 7.90]	2019
**Subtotal (95% CI)**			**25.6%**	**1.48 [1.21, 1.76]**	
Heterogeneity: Tau² = 0.03; Chi² = 6.07, df = 2 (P = 0.05); I² = 67%					
Test for overall effect: Z = 10.65 (P < 0.00001)					
**1.1.3 Case-control study**					
Park 2005	0.2	1.072	2.6%	0.20 [-1.90, 2.30]	2005
Kebapcilar 2010	-2.2	3.322	0.3%	-2.20 [-8.71, 4.31]	2010
Cornejo-Pareja 2019	2.4	0.531	6.6%	2.40 [1.36, 3.44]	2019
**Subtotal (95% CI)**			**9.5%**	**1.15 [-0.95, 3.26]**	
Heterogeneity: Tau² = 1.89; Chi² = 4.92, df = 2 (P = 0.09); I² = 59%					
Test for overall effect: Z = 1.07 (P = 0.28)					
**1.1.4 Before-after study**					
De Luis 1999	5.5	5.266	0.1%	5.50 [-4.82, 15.82]	1999
Schweeger 2000	0	0.7449	4.5%	0.00 [-1.46, 1.46]	2000
Elizalde 2002	2.32	0.03	12.8%	2.32 [2.26, 2.38]	2002
Lu 2002	4.2	3.3	0.3%	4.20 [-2.27, 10.67]	2002
Scharnagl 2004	6	1.674	1.2%	6.00 [2.72, 9.28]	2004
Kanbay 2005	2	2.035	0.9%	2.00 [-1.99, 5.99]	2005
Pellicano 2009	4	0.6899	4.9%	4.00 [2.65, 5.35]	2009
Gen 2010	6	1.962	0.9%	6.00 [2.15, 9.85]	2010
Ando 2013	2.5	0.119	12.2%	2.50 [2.27, 2.73]	2013
Mokhtare 2017	2.69	0.668	5.1%	2.69 [1.38, 4.00]	2017
Iwai 2019	2.1	1.69	1.2%	2.10 [-1.21, 5.41]	2019
**Subtotal (95% CI)**			**44.2%**	**2.49 [2.07, 2.90]**	
Heterogeneity: Tau² = 0.12; Chi² = 27.11, df = 10 (P = 0.003); I² = 63%					
Test for overall effect: Z = 11.77 (P < 0.00001)					
**Total (95% CI)**			**100.0%**	**2.28 [1.90, 2.66]**	
Heterogeneity: Tau² = 0.30; Chi² = 604.11, df = 20 (P < 0.00001); I² = 97%					
Test for overall effect: Z = 11.73 (P < 0.00001)					
Test for subgroup differences: Chi² = 16.78, df = 3 (P = 0.0008), I² = 82.1%					

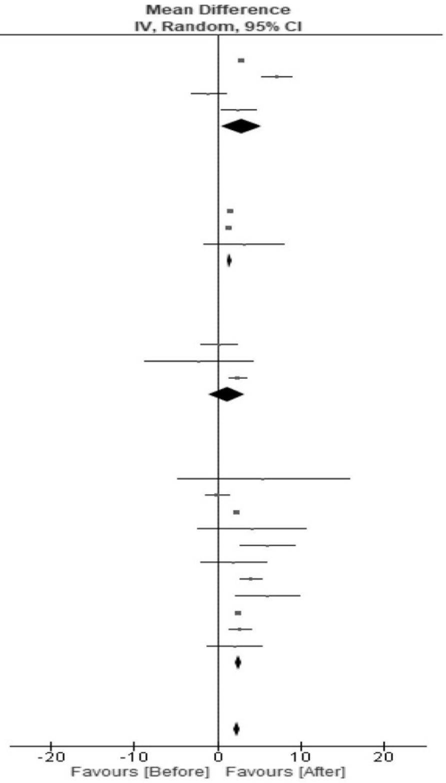

Supplementary Figure S3. Forest plot of the mean changes in high-density lipoprotein cholesterol levels before and after *Helicobacter pylori* eradication for each study design

Study or Subgroup	Mean Difference	SE	Weight	Mean Difference IV, Random, 95% CI	Year
**1.2.1 Randomized control trial**					
Jamali 2013	-54.9	16.434	0.4%	-54.90 [-87.11, -22.69]	2013
Mirmiran 2014	23	27.0414	0.2%	23.00 [-30.00, 76.00]	2014
Liou 2019	-15.66	4.347	4.0%	-15.66 [-24.18, -7.14]	2019
**Subtotal (95% CI)**			**4.6%**	**-20.24 [-53.16, 12.68]**	
Heterogeneity: Tau² = 593.72; Chi² = 7.60, df = 2 (P = 0.02); I² = 74%					
Test for overall effect: Z = 1.21 (P = 0.23)					
**1.2.2 Cohort study**					
Furuta 2002	6.4	0.6014	11.8%	6.40 [5.22, 7.58]	2002
Kamada 2005	48.8	27.5796	0.1%	48.80 [-5.26, 102.86]	2005
Nam 2015	12.5	0.152	12.2%	12.50 [12.20, 12.80]	2015
Adachi 2018	1.2	0.8074	11.5%	1.20 [-0.38, 2.78]	2018
Huang 2019	-7.15	7.6429	1.7%	-7.15 [-22.13, 7.83]	2019
**Subtotal (95% CI)**			**37.3%**	**5.75 [-0.52, 12.02]**	
Heterogeneity: Tau² = 34.62; Chi² = 282.11, df = 4 (P < 0.00001); I² = 99%					
Test for overall effect: Z = 1.80 (P = 0.07)					
**1.2.3 Case-control study**					
Park 2005	2.5	8.052	1.5%	2.50 [-13.28, 18.28]	2005
Kebapcilar 2010	12.4	10.602	0.9%	12.40 [-8.38, 33.18]	2010
Cornejo-Pareja 2019	2.4	0.531	11.9%	2.40 [1.36, 3.44]	2019
**Subtotal (95% CI)**			**14.4%**	**2.43 [1.39, 3.46]**	
Heterogeneity: Tau² = 0.00; Chi² = 0.89, df = 2 (P = 0.64); I² = 0%					
Test for overall effect: Z = 4.58 (P < 0.00001)					
**1.2.4 Before-after study**					
De Luis 1999	-0.4	18.674	0.3%	-0.40 [-37.00, 36.20]	1999
Schweeger 2000	1.58	5.944	2.5%	1.58 [-10.07, 13.23]	2000
Elizalde 2002	8.857	0.002	12.3%	8.86 [8.85, 8.86]	2002
Lu 2002	-9.6	11.026	0.9%	-9.60 [-31.21, 12.01]	2002
Scharnagl 2004	20	9.885	1.1%	20.00 [0.63, 39.37]	2004
Kanbay 2005	2.5	7.912	1.6%	2.50 [-13.01, 18.01]	2005
Pellicano 2009	-3	3.4299	5.4%	-3.00 [-9.72, 3.72]	2009
Gen 2010	-30	13.204	0.6%	-30.00 [-55.88, -4.12]	2010
Ando 2013	3.3	0.497	12.0%	3.30 [2.33, 4.27]	2013
Mokhtare 2017	-5.54	3.94	4.6%	-5.54 [-13.26, 2.18]	2017
Iwai 2019	5.4	6.044	2.5%	5.40 [-6.45, 17.25]	2019
**Subtotal (95% CI)**			**43.7%**	**2.07 [-1.88, 6.03]**	
Heterogeneity: Tau² = 18.13; Chi² = 165.77, df = 10 (P < 0.00001); I² = 94%					
Test for overall effect: Z = 1.03 (P = 0.30)					
**Total (95% CI)**			**100.0%**	**3.22 [1.13, 5.31]**	
Heterogeneity: Tau² = 9.26; Chi² = 1049.01, df = 21 (P < 0.00001); I² = 98%					
Test for overall effect: Z = 3.02 (P = 0.003)					
Test for subgroup differences: Chi² = 2.93, df = 3 (P = 0.40), I² = 0%					

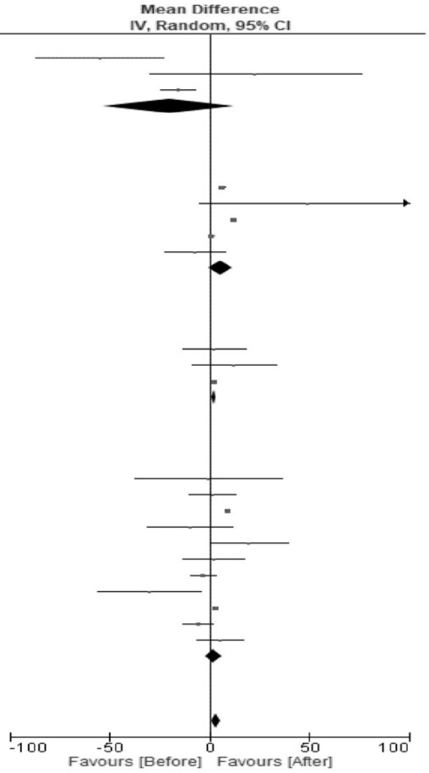

Supplementary Figure S4. Forest plot of the mean changes in triglyceride levels before and after *Helicobacter pylori* eradication for each study design

Journal of
Clinical Medicine

Study or Subgroup	Mean Difference	SE	Weight	Mean Difference IV, Random, 95% CI	Year
**1.3.1 Randomized control trial**					
Zhang 2006	-0.4	0.3795	6.7%	-0.40 [-1.14, 0.34]	2006
Jamali 2013	-4.7	5.007	3.4%	-4.70 [-14.51, 5.11]	2013
Mirmiran 2014	4	8.345	1.8%	4.00 [-12.36, 20.36]	2014
Liou 2019	2.17	1.3521	6.3%	2.17 [-0.48, 4.82]	2019
**Subtotal (95% CI)**			**18.3%**	**0.27 [-1.61, 2.15]**	
Heterogeneity: Tau² = 1.26; Chi² = 4.41, df = 3 (P = 0.22); I² = 32%					
Test for overall effect: Z = 0.28 (P = 0.78)					
**1.3.2 Cohort study**					
Nam 2015	-6.4	0.048	6.8%	-6.40 [-6.49, -6.31]	2015
Adachi 2018	-0.9	0.2056	6.8%	-0.90 [-1.30, -0.50]	2018
Huang 2019	-17.14	4.4664	3.8%	-17.14 [-25.89, -8.39]	2019
**Subtotal (95% CI)**			**17.4%**	**-6.05 [-10.95, -1.15]**	
Heterogeneity: Tau² = 15.18; Chi² = 684.72, df = 2 (P < 0.00001); I² = 100%					
Test for overall effect: Z = 2.42 (P = 0.02)					
**1.3.3 Case-control study**					
Park 2005	0.7	2.543	5.4%	0.70 [-4.28, 5.68]	2005
Kebapcilar 2010	-7.2	8.305	1.8%	-7.20 [-23.48, 9.08]	2010
Cornejo-Pareja 2019	2.4	0.531	6.7%	2.40 [1.36, 3.44]	2019
**Subtotal (95% CI)**			**14.0%**	**2.29 [1.28, 3.31]**	
Heterogeneity: Tau² = 0.00; Chi² = 1.74, df = 2 (P = 0.42); I² = 0%					
Test for overall effect: Z = 4.42 (P < 0.00001)					
**1.3.4 Before-after study**					
De Luis 1999	-10.2	8.309	1.8%	-10.20 [-26.49, 6.09]	1999
Schweeger 2000	1.33	2.4949	5.5%	1.33 [-3.56, 6.22]	2000
Lu 2002	5.3	7.025	2.3%	5.30 [-8.47, 19.07]	2002
Elizalde 2002	1.547	0.071	6.8%	1.55 [1.41, 1.69]	2002
Scharnagl 2004	6	7.126	2.3%	6.00 [-7.97, 19.97]	2004
Kanbay 2005	-1.1	6.382	2.6%	-1.10 [-13.61, 11.41]	2005
Pellicano 2009	-3	2.1853	5.7%	-3.00 [-7.28, 1.28]	2009
Gen 2010	-15	2.188	5.7%	-15.00 [-19.29, -10.71]	2010
Ando 2013	0.3	0.183	6.8%	0.30 [-0.06, 0.66]	2013
Mokhtare 2017	-6.23	1.887	6.0%	-6.23 [-9.93, -2.53]	2017
Iwai 2019	-2.2	3.119	4.9%	-2.20 [-8.31, 3.91]	2019
**Subtotal (95% CI)**			**50.4%**	**-1.65 [-3.14, -0.15]**	
Heterogeneity: Tau² = 2.06; Chi² = 120.67, df = 10 (P < 0.00001); I² = 92%					
Test for overall effect: Z = 2.16 (P = 0.03)					
**Total (95% CI)**			**100.0%**	**-2.33 [-4.92, 0.26]**	
Heterogeneity: Tau² = 25.76; Chi² = 9550.85, df = 20 (P < 0.00001); I² = 100%					
Test for overall effect: Z = 1.76 (P = 0.08)					
Test for subgroup differences: Chi² = 26.34, df = 3 (P < 0.00001), I² = 88.6%					

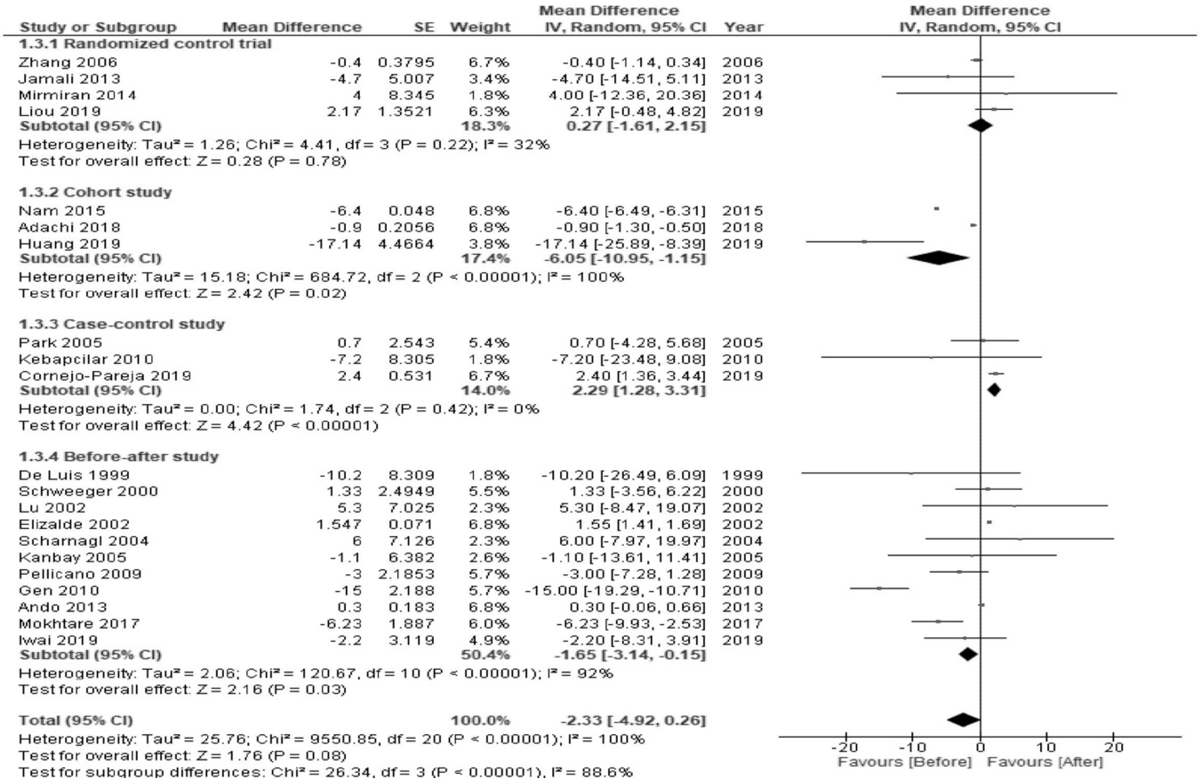

Supplementary Figure S5. Forest plot of the mean changes in low-density lipoprotein cholesterol levels before and after *Helicobacter pylori* eradication for each study design

Study or Subgroup	Mean Difference	SE	Weight	Mean Difference IV, Random, 95% CI	Year
**1.4.1 Randomized control trial**					
Zhang 2006	-1.5	0.3305	6.8%	-1.50 [-2.15, -0.85]	2006
Jamali 2013	-13.6	5.497	4.1%	-13.60 [-24.37, -2.83]	2013
Mirmiran 2014	8	11.035	1.8%	8.00 [-13.63, 29.63]	2014
Liou 2019	1.61	1.5153	6.5%	1.61 [-1.36, 4.58]	2019
**Subtotal (95% CI)**			**19.2%**	**-1.23 [-4.99, 2.53]**	
Heterogeneity: Tau² = 7.35; Chi² = 9.69, df = 3 (P = 0.02); I² = 69%					
Test for overall effect: Z = 0.64 (P = 0.52)					
**1.4.2 Cohort study**					
Furuta 2002	6.3	0.1972	6.8%	6.30 [5.91, 6.69]	2002
Azuma 2002	3.3	20.4929	0.6%	3.30 [-36.87, 43.47]	2002
Kamada 2005	17.1	7.2217	3.1%	17.10 [2.95, 31.25]	2005
Nam 2015	-5.1	0.052	6.8%	-5.10 [-5.20, -5.00]	2015
Huang 2019	-12.4	8.7394	2.5%	-12.40 [-29.53, 4.73]	2019
**Subtotal (95% CI)**			**20.0%**	**1.72 [-7.18, 10.63]**	
Heterogeneity: Tau² = 65.01; Chi² = 3134.47, df = 4 (P < 0.00001); I² = 100%					
Test for overall effect: Z = 0.38 (P = 0.70)					
**1.4.3 Case-control study**					
Park 2005	4.6	2.916	5.7%	4.60 [-1.12, 10.32]	2005
Kebapcilar 2010	-7.4	9.059	2.4%	-7.40 [-25.16, 10.36]	2010
Cornejo-Pareja 2019	-2.9	1.431	6.5%	-2.90 [-5.70, -0.10]	2019
**Subtotal (95% CI)**			**14.7%**	**-0.46 [-6.72, 5.80]**	
Heterogeneity: Tau² = 17.91; Chi² = 5.76, df = 2 (P = 0.06); I² = 65%					
Test for overall effect: Z = 0.14 (P = 0.89)					
**1.4.4 Before-after study**					
De Luis 1999	-4.4	10.5698	1.9%	-4.40 [-25.12, 16.32]	1999
Schweeger 2000	3.39	2.6888	5.9%	3.39 [-1.88, 8.66]	2000
Lu 2002	-0.5	8.6316	2.5%	-0.50 [-17.42, 16.42]	2002
Elizalde 2002	5.027	0.071	6.8%	5.03 [4.89, 5.17]	2002
Scharnagl 2004	21	7.657	2.9%	21.00 [5.99, 36.01]	2004
Kanbay 2005	-3	6.445	3.5%	-3.00 [-15.63, 9.63]	2005
Gen 2010	-24	4.94	4.4%	-24.00 [-33.68, -14.32]	2010
Ando 2013	2.4	0.2128	6.8%	2.40 [1.98, 2.82]	2013
Mokhtare 2017	-8.64	2.623	5.9%	-8.64 [-13.78, -3.50]	2017
Iwai 2019	-0.9	3.507	5.3%	-0.90 [-7.77, 5.97]	2019
**Subtotal (95% CI)**			**46.2%**	**0.57 [-1.73, 2.87]**	
Heterogeneity: Tau² = 4.88; Chi² = 207.02, df = 9 (P < 0.00001); I² = 96%					
Test for overall effect: Z = 0.48 (P = 0.63)					
**Total (95% CI)**			**100.0%**	**-0.67 [-4.07, 2.72]**	
Heterogeneity: Tau² = 43.89; Chi² = 15108.32, df = 21 (P < 0.00001); I² = 100%					
Test for overall effect: Z = 0.39 (P = 0.70)					
Test for subgroup differences: Chi² = 0.80, df = 3 (P = 0.85), I² = 0%					

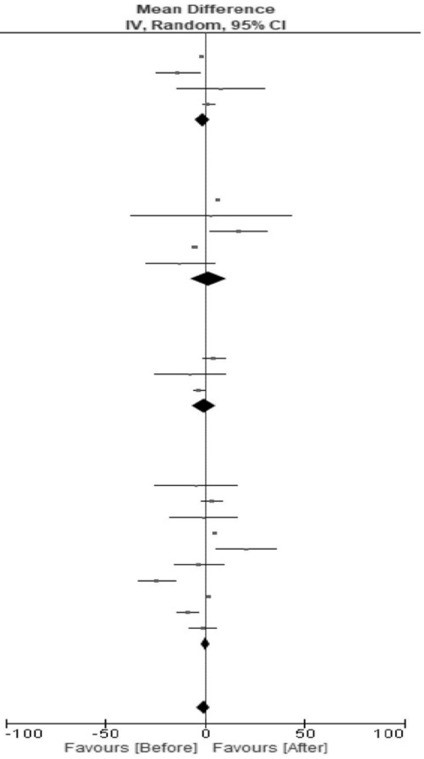

Supplementary Figure S6. Forest plot of the mean changes in total cholesterol levels before and after *Helicobacter pylori* eradication for each study design

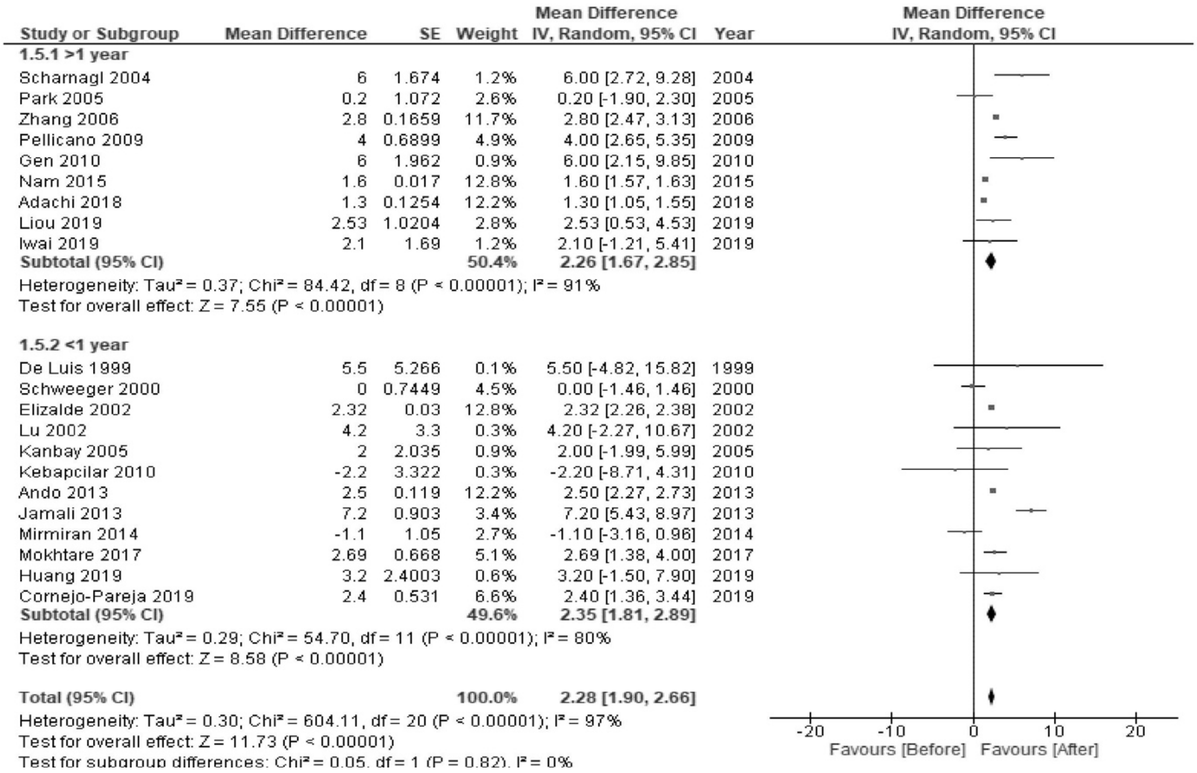

Supplementary Figure S7. Forest plot of the mean changes in high-density lipoprotein cholesterol levels before and after *Helicobacter pylori* eradication during < 1- or ≥ 1-year follow-up

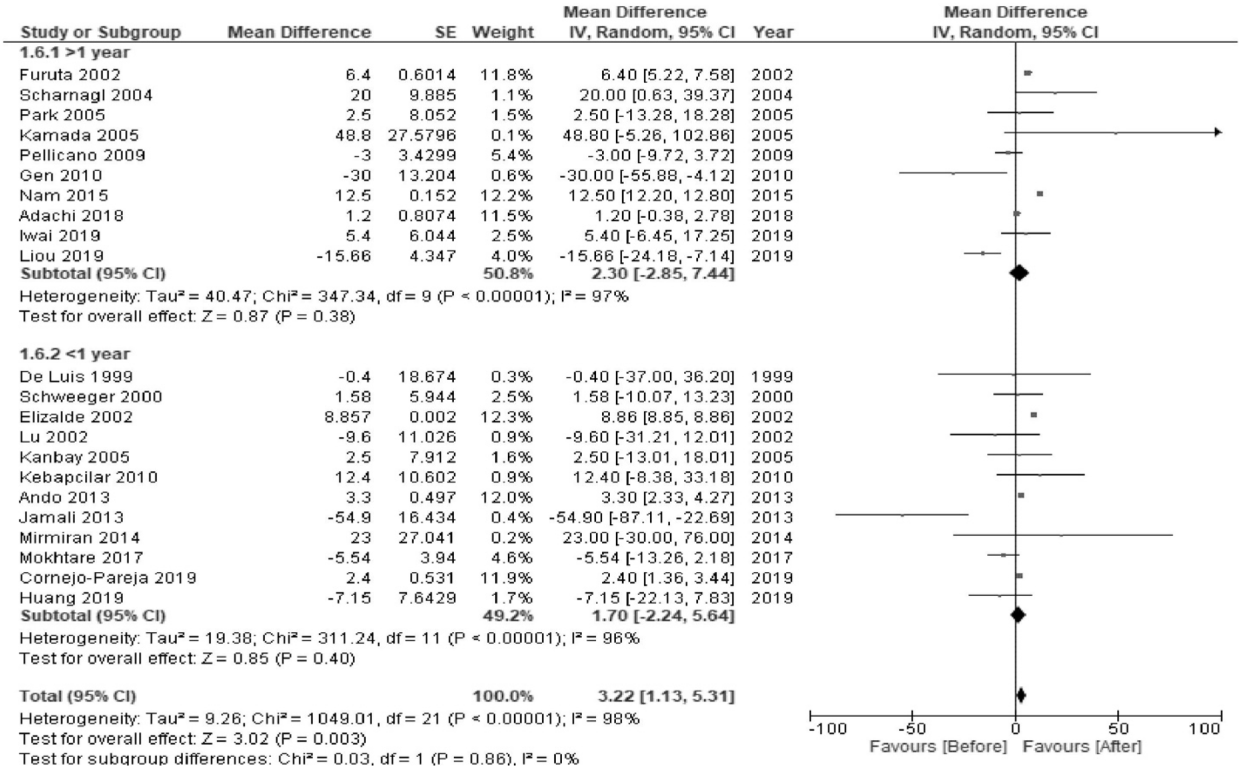

Supplementary Figure S8. Forest plot of the mean changes in triglyceride levels before and after *Helicobacter pylori* eradication during < 1- or ≥ 1-year follow-up

Journal of
Clinical Medicine

MDPI

Study or Subgroup	Mean Difference	SE	Weight	Mean Difference IV, Random, 95% CI	Year
**1.7.1 >1 year**					
Scharnagl 2004	6	7.126	2.3%	6.00 [-7.97, 19.97]	2004
Park 2005	0.7	2.543	5.4%	0.70 [-4.28, 5.68]	2005
Zhang 2006	-0.4	0.3795	6.7%	-0.40 [-1.14, 0.34]	2006
Pellicano 2009	-3	2.1853	5.7%	-3.00 [-7.28, 1.28]	2009
Gen 2010	-15	2.188	5.7%	-15.00 [-19.29, -10.71]	2010
Nam 2015	-6.4	0.048	6.8%	-6.40 [-6.49, -6.31]	2015
Adachi 2018	-0.9	0.2056	6.8%	-0.90 [-1.30, -0.50]	2018
Liou 2019	2.17	1.3521	6.3%	2.17 [-0.48, 4.82]	2019
Iwai 2019	-2.2	3.119	4.9%	-2.20 [-8.31, 3.91]	2019
**Subtotal (95% CI)**			**50.6%**	**-2.72 [-5.68, 0.24]**	
Heterogeneity: Tau² = 15.96; Chi² = 968.90, df = 8 (P < 0.00001); I² = 99%					
Test for overall effect: Z = 1.80 (P = 0.07)					
**1.7.2 <1 year**					
De Luis 1999	-10.28	8.309	1.8%	-10.28 [-26.57, 6.01]	1999
Schweeger 2000	1.33	2.4949	5.5%	1.33 [-3.56, 6.22]	2000
Elizalde 2002	1.547	0.071	6.8%	1.55 [1.41, 1.69]	2002
Lu 2002	5.37	7.025	2.3%	5.37 [-8.40, 19.14]	2002
Kanbay 2005	-1.1	6.382	2.6%	-1.10 [-13.61, 11.41]	2005
Kebapcilar 2010	-7.2	8.305	1.8%	-7.20 [-23.48, 9.08]	2010
Ando 2013	0.3	0.183	6.8%	0.30 [-0.06, 0.66]	2013
Jamali 2013	-4.7	5.007	3.4%	-4.70 [-14.51, 5.11]	2013
Mirmiran 2014	4	8.345	1.8%	4.00 [-12.36, 20.36]	2014
Mokhtare 2017	-6.23	1.887	6.0%	-6.23 [-9.93, -2.53]	2017
Cornejo-Pareja 2019	2.4	0.531	6.7%	2.40 [1.36, 3.44]	2019
Huang 2019	-17.14	4.4664	3.8%	-17.14 [-25.89, -8.39]	2019
**Subtotal (95% CI)**			**49.4%**	**0.32 [-0.85, 1.49]**	
Heterogeneity: Tau² = 1.21; Chi² = 82.59, df = 11 (P < 0.00001); I² = 87%					
Test for overall effect: Z = 0.53 (P = 0.59)					
**Total (95% CI)**			**100.0%**	**-2.33 [-4.92, 0.26]**	
Heterogeneity: Tau² = 25.76; Chi² = 9550.89, df = 20 (P < 0.00001); I² = 100%					
Test for overall effect: Z = 1.76 (P = 0.08)					
Test for subgroup differences: Chi² = 3.50, df = 1 (P = 0.06), I² = 71.4%					

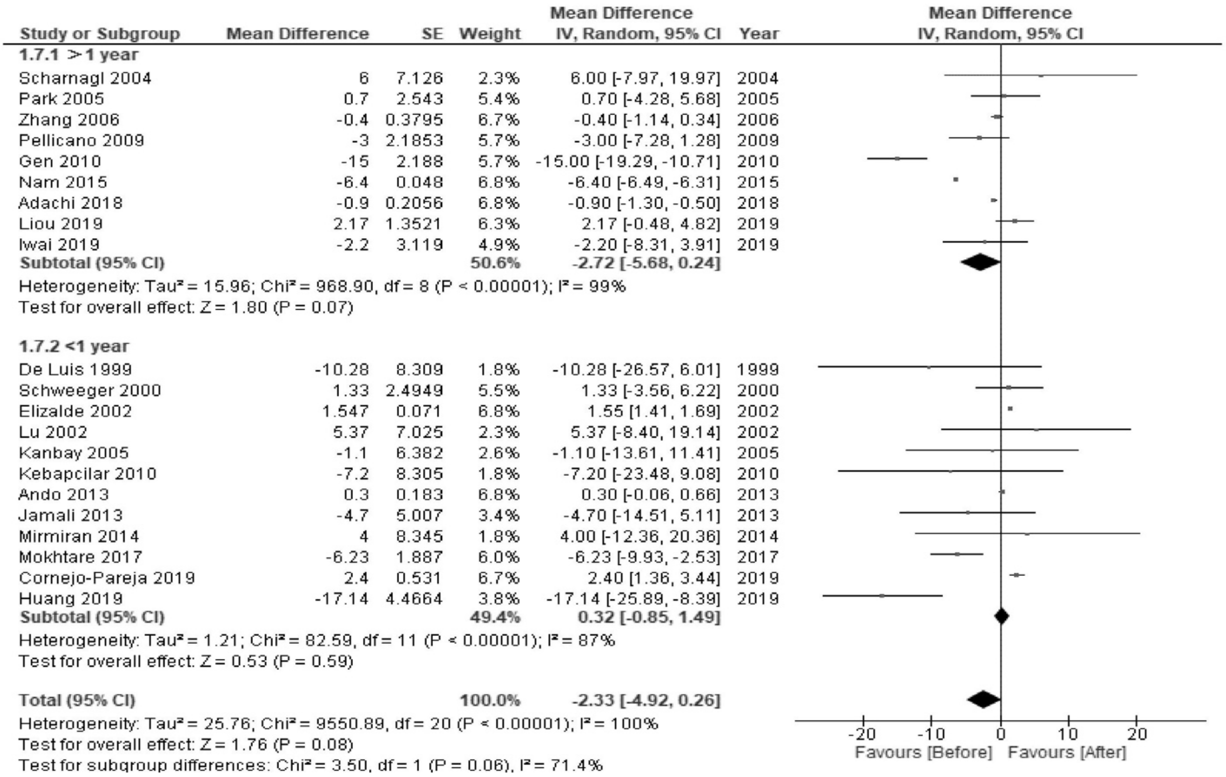

Supplementary Figure S9. Forest plot of the mean changes in low-density lipoprotein cholesterol levels before and after *Helicobacter pylori* eradication during < 1- or ≥ 1-year follow-up

Study or Subgroup	Mean Difference	SE	Weight	Mean Difference IV, Random, 95% CI	Year
**1.8.1 >1 year**					
Azuma 2002	3.3	20.4929	0.6%	3.30 [-36.87, 43.47]	2002
Furuta 2002	6.3	0.1972	6.8%	6.30 [5.91, 6.69]	2002
Scharnagl 2004	21	7.657	2.9%	21.00 [5.99, 36.01]	2004
Kamada 2005	17.1	7.2217	3.1%	17.10 [2.95, 31.25]	2005
Park 2005	4.6	2.916	5.7%	4.60 [-1.12, 10.32]	2005
Zhang 2006	-1.5	0.3305	6.8%	-1.50 [-2.15, -0.85]	2006
Gen 2010	-24	4.94	4.4%	-24.00 [-33.68, -14.32]	2010
Nam 2015	-5.1	0.052	6.8%	-5.10 [-5.20, -5.00]	2015
Iwai 2019	-0.9	3.507	5.3%	-0.90 [-7.77, 5.97]	2019
Liou 2019	1.61	1.5153	6.5%	1.61 [-1.36, 4.58]	2019
**Subtotal (95% CI)**			**49.2%**	**0.90 [-4.17, 5.98]**	
Heterogeneity: Tau² = 49.09; Chi² = 3258.90, df = 9 (P < 0.00001); I² = 100%					
Test for overall effect: Z = 0.35 (P = 0.73)					
**1.8.2 <1 year**					
De Luis 1999	-4.4	10.57	1.9%	-4.40 [-25.12, 16.32]	1999
Schweeger 2000	3.39	2.6888	5.9%	3.39 [-1.88, 8.66]	2000
Elizalde 2002	5.027	0.071	6.8%	5.03 [4.89, 5.17]	2002
Lu 2002	-0.5	8.632	2.5%	-0.50 [-17.42, 16.42]	2002
Kanbay 2005	-3	6.445	3.5%	-3.00 [-15.63, 9.63]	2005
Kebapcilar 2010	-7.4	9.059	2.4%	-7.40 [-25.16, 10.36]	2010
Ando 2013	2.4	0.2128	6.8%	2.40 [1.98, 2.82]	2013
Jamali 2013	-13.6	5.497	4.1%	-13.60 [-24.37, -2.83]	2013
Mirmiran 2014	8	11.035	1.8%	8.00 [-13.63, 29.63]	2014
Mokhtare 2017	-8.64	2.623	5.9%	-8.64 [-13.78, -3.50]	2017
Huang 2019	-12.4	8.7394	2.5%	-12.40 [-29.53, 4.73]	2019
Cornejo-Pareja 2019	-2.9	1.431	6.5%	-2.90 [-5.70, -0.10]	2019
**Subtotal (95% CI)**			**50.8%**	**-0.03 [-2.18, 2.13]**	
Heterogeneity: Tau² = 4.85; Chi² = 211.44, df = 11 (P < 0.00001); I² = 95%					
Test for overall effect: Z = 0.02 (P = 0.98)					
**Total (95% CI)**			**100.0%**	**-0.67 [-4.07, 2.72]**	
Heterogeneity: Tau² = 43.89; Chi² = 15108.32, df = 21 (P < 0.00001); I² = 100%					
Test for overall effect: Z = 0.39 (P = 0.70)					
Test for subgroup differences: Chi² = 0.11, df = 1 (P = 0.74), I² = 0%					

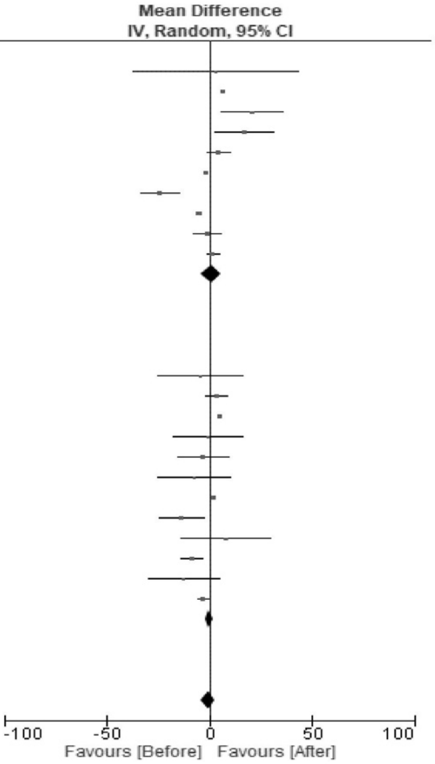

Supplementary Figure S10. Forest plot of the mean changes in total cholesterol levels before and after *Helicobacter pylori* eradication during < 1- or ≥ 1-year follow-up

Journal of
Clinical Medicine

Study or Subgroup	Mean Difference	SE	Weight	Mean Difference IV, Random, 95% CI	Year
**1.9.1 Western countries**					
De Luis 1999	5.5	5.266	0.1%	5.50 [-4.82, 15.82]	1999
Schweeger 2000	0	0.7449	4.5%	0.00 [-1.46, 1.46]	2000
Elizalde 2002	2.32	0.03	12.8%	2.32 [2.26, 2.38]	2002
Scharnagl 2004	6	1.674	1.2%	6.00 [2.72, 9.28]	2004
Kanbay 2005	2	2.035	0.9%	2.00 [-1.99, 5.99]	2005
Pellicano 2009	4	0.6899	4.9%	4.00 [2.65, 5.35]	2009
Gen 2010	6	1.962	0.9%	6.00 [2.15, 9.85]	2010
Kebapcilar 2010	-2.2	3.322	0.3%	-2.20 [-8.71, 4.31]	2010
Jamali 2013	7.2	0.903	3.4%	7.20 [5.43, 8.97]	2013
Mirmiran 2014	-1.1	1.05	2.7%	-1.10 [-3.16, 0.96]	2014
Mokhtare 2017	2.69	0.668	5.1%	2.69 [1.38, 4.00]	2017
Cornejo-Pareja 2019	2.4	0.531	6.6%	2.40 [1.36, 3.44]	2019
**Subtotal (95% CI)**			**43.4%**	**2.81 [1.73, 3.90]**	
Heterogeneity: Tau² = 2.17; Chi² = 66.34, df = 11 (P < 0.00001); I² = 83%					
Test for overall effect: Z = 5.08 (P < 0.00001)					
**1.9.2 Asia**					
Lu 2002	4.2	3.3	0.3%	4.20 [-2.27, 10.67]	2002
Park 2005	0.2	1.072	2.6%	0.20 [-1.90, 2.30]	2005
Zhang 2006	2.8	0.1659	11.7%	2.80 [2.47, 3.13]	2006
Ando 2013	2.5	0.119	12.2%	2.50 [2.27, 2.73]	2013
Nam 2015	1.6	0.017	12.8%	1.60 [1.57, 1.63]	2015
Adachi 2018	1.3	0.1254	12.2%	1.30 [1.05, 1.55]	2018
Iwai 2019	2.1	1.69	1.2%	2.10 [-1.21, 5.41]	2019
Huang 2019	3.2	2.4003	0.6%	3.20 [-1.50, 7.90]	2019
Liou 2019	2.53	1.0204	2.8%	2.53 [0.53, 4.53]	2019
**Subtotal (95% CI)**			**56.6%**	**2.00 [1.48, 2.53]**	
Heterogeneity: Tau² = 0.32; Chi² = 116.78, df = 8 (P < 0.00001); I² = 93%					
Test for overall effect: Z = 7.44 (P < 0.00001)					
**Total (95% CI)**			**100.0%**	**2.28 [1.90, 2.66]**	
Heterogeneity: Tau² = 0.30; Chi² = 604.11, df = 20 (P < 0.00001); I² = 97%					
Test for overall effect: Z = 11.73 (P < 0.00001)					
Test for subgroup differences: Chi² = 1.73, df = 1 (P = 0.19), I² = 42.2%					

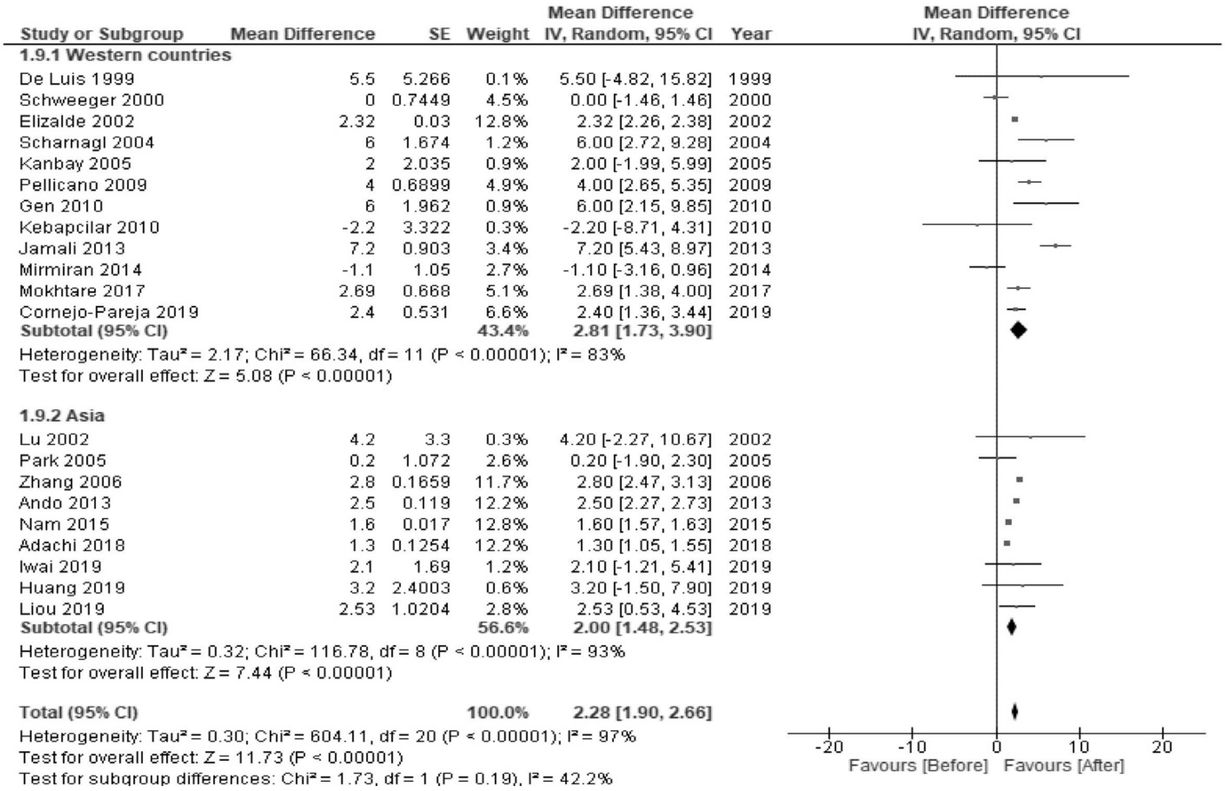

Supplementary Figure S11. Forest plot of the mean changes in high-density lipoprotein cholesterol levels before and after *Helicobacter pylori* eradication in Western countries or Asia

Study or Subgroup	Mean Difference	SE	Weight	Mean Difference IV, Random, 95% CI	Year
**1.10.1 Western countries**					
De Luis 1999	-0.4	18.674	0.3%	-0.40 [-37.00, 36.20]	1999
Schweeger 2000	1.58	5.944	2.5%	1.58 [-10.07, 13.23]	2000
Elizalde 2002	8.857	0.002	12.3%	8.86 [8.85, 8.86]	2002
Scharnagl 2004	20	9.885	1.1%	20.00 [0.63, 39.37]	2004
Kanbay 2005	2.5	7.912	1.6%	2.50 [-13.01, 18.01]	2005
Pellicano 2009	-3	3.4299	5.4%	-3.00 [-9.72, 3.72]	2009
Kebapcilar 2010	12.4	10.602	0.9%	12.40 [-8.38, 33.18]	2010
Gen 2010	-30	13.204	0.6%	-30.00 [-55.88, -4.12]	2010
Jamali 2013	-54.9	16.434	0.4%	-54.90 [-87.11, -22.69]	2013
Mirmiran 2014	23	27.041	0.2%	23.00 [-30.00, 76.00]	2014
Mokhtare 2017	-5.54	3.94	4.6%	-5.54 [-13.26, 2.18]	2017
Cornejo-Pareja 2019	2.4	0.531	11.9%	2.40 [1.36, 3.44]	2019
**Subtotal (95% CI)**			**41.8%**	**1.23 [-3.31, 5.77]**	
Heterogeneity: Tau² = 25.17; Chi² = 200.92, df = 11 (P < 0.00001); I² = 95%					
Test for overall effect: Z = 0.53 (P = 0.60)					
**1.10.2 Asia**					
Lu 2002	-9.6	11.026	0.9%	-9.60 [-31.21, 12.01]	2002
Furuta 2002	6.4	0.6014	11.8%	6.40 [5.22, 7.58]	2002
Kamada 2005	48.8	27.5796	0.1%	48.80 [-5.26, 102.86]	2005
Park 2005	2.5	8.052	1.5%	2.50 [-13.28, 18.28]	2005
Ando 2013	3.3	0.497	12.0%	3.30 [2.33, 4.27]	2013
Nam 2015	12.5	0.152	12.2%	12.50 [12.20, 12.80]	2015
Adachi 2018	1.2	0.8074	11.5%	1.20 [-0.38, 2.78]	2018
Liou 2019	-15.66	4.347	4.0%	-15.66 [-24.18, -7.14]	2019
Iwai 2019	5.4	6.044	2.5%	5.40 [-6.45, 17.25]	2019
Huang 2019	-7.15	7.6429	1.7%	-7.15 [-22.13, 7.83]	2019
**Subtotal (95% CI)**			**58.2%**	**2.20 [-2.70, 7.10]**	
Heterogeneity: Tau² = 38.86; Chi² = 593.55, df = 9 (P < 0.00001); I² = 98%					
Test for overall effect: Z = 0.88 (P = 0.38)					
**Total (95% CI)**			**100.0%**	**3.22 [1.13, 5.31]**	
Heterogeneity: Tau² = 9.26; Chi² = 1049.01, df = 21 (P < 0.00001); I² = 98%					
Test for overall effect: Z = 3.02 (P = 0.003)					
Test for subgroup differences: Chi² = 0.08, df = 1 (P = 0.78), I² = 0%					

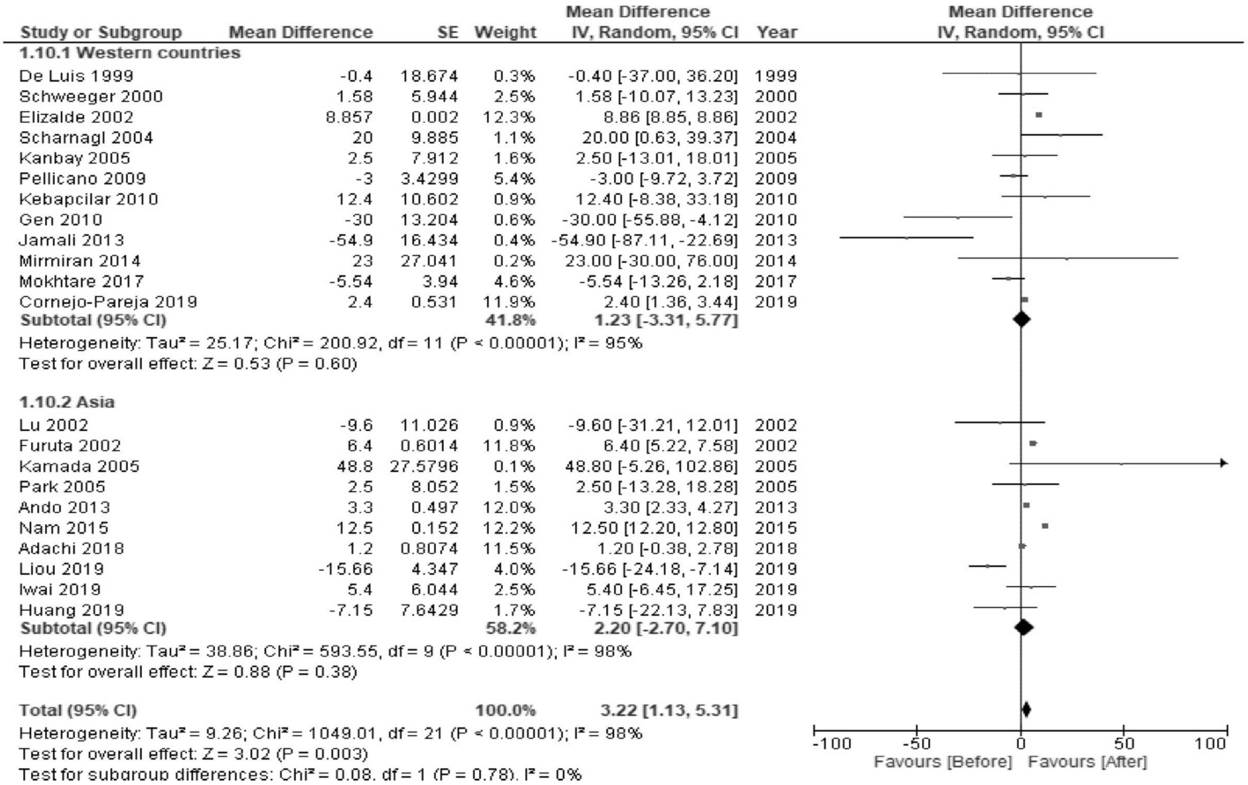

Supplementary Figure S12. Forest plot of the mean changes in triglyceride levels before and after *Helicobacter pylori* eradication in Western countries or Asia

Journal of
Clinical Medicine

MDPI

Study or Subgroup	Mean Difference	SE	Weight	Mean Difference IV, Random, 95% CI	Year
**1.11.1 Western countries**					
De Luis 1999	-10.2	8.309	1.8%	-10.20 [-26.49, 6.09]	1999
Schweeger 2000	1.33	2.4949	5.5%	1.33 [-3.56, 6.22]	2000
Elizalde 2002	1.547	0.071	6.8%	1.55 [1.41, 1.69]	2002
Scharnagl 2004	6	7.126	2.3%	6.00 [-7.97, 19.97]	2004
Kanbay 2005	-1.1	6.382	2.6%	-1.10 [-13.61, 11.41]	2005
Pellicano 2009	-3	2.1853	5.7%	-3.00 [-7.28, 1.28]	2009
Kebapcilar 2010	-7.2	8.305	1.8%	-7.20 [-23.48, 9.08]	2010
Gen 2010	-15	2.188	5.7%	-15.00 [-19.29, -10.71]	2010
Jamali 2013	-4.7	5.007	3.4%	-4.70 [-14.51, 5.11]	2013
Mirmiran 2014	4	8.345	1.8%	4.00 [-12.36, 20.36]	2014
Mokhtare 2017	-6.23	1.887	6.0%	-6.23 [-9.93, -2.53]	2017
Cornejo-Pareja 2019	2.4	0.531	6.7%	2.40 [1.36, 3.44]	2019
**Subtotal (95% CI)**			**50.2%**	**-2.44 [-4.89, 0.01]**	
Heterogeneity: Tau² = 8.46; Chi² = 86.33, df = 11 (P < 0.00001); I² = 87%					
Test for overall effect: Z = 1.95 (P = 0.05)					
**1.11.2 Asia**					
Lu 2002	5.3	7.025	2.3%	5.30 [-8.47, 19.07]	2002
Park 2005	0.7	2.543	5.4%	0.70 [-4.28, 5.68]	2005
Zhang 2006	-0.4	0.3795	6.7%	-0.40 [-1.14, 0.34]	2006
Ando 2013	0.3	0.183	6.8%	0.30 [-0.06, 0.66]	2013
Nam 2015	-6.4	0.048	6.8%	-6.40 [-6.49, -6.31]	2015
Adachi 2018	-0.9	0.2056	6.8%	-0.90 [-1.30, -0.50]	2018
Huang 2019	-17.14	4.4664	3.8%	-17.14 [-25.89, -8.39]	2019
Iwai 2019	-2.2	3.119	4.9%	-2.20 [-8.31, 3.91]	2019
Liou 2019	2.17	1.3521	6.3%	2.17 [-0.48, 4.82]	2019
**Subtotal (95% CI)**			**49.8%**	**-1.86 [-5.04, 1.31]**	
Heterogeneity: Tau² = 18.46; Chi² = 2076.29, df = 8 (P < 0.00001); I² = 100%					
Test for overall effect: Z = 1.15 (P = 0.25)					
**Total (95% CI)**			**100.0%**	**-2.33 [-4.92, 0.26]**	
Heterogeneity: Tau² = 25.76; Chi² = 9550.85, df = 20 (P < 0.00001); I² = 100%					
Test for overall effect: Z = 1.76 (P = 0.08)					
Test for subgroup differences: Chi² = 0.08, df = 1 (P = 0.78), I² = 0%					

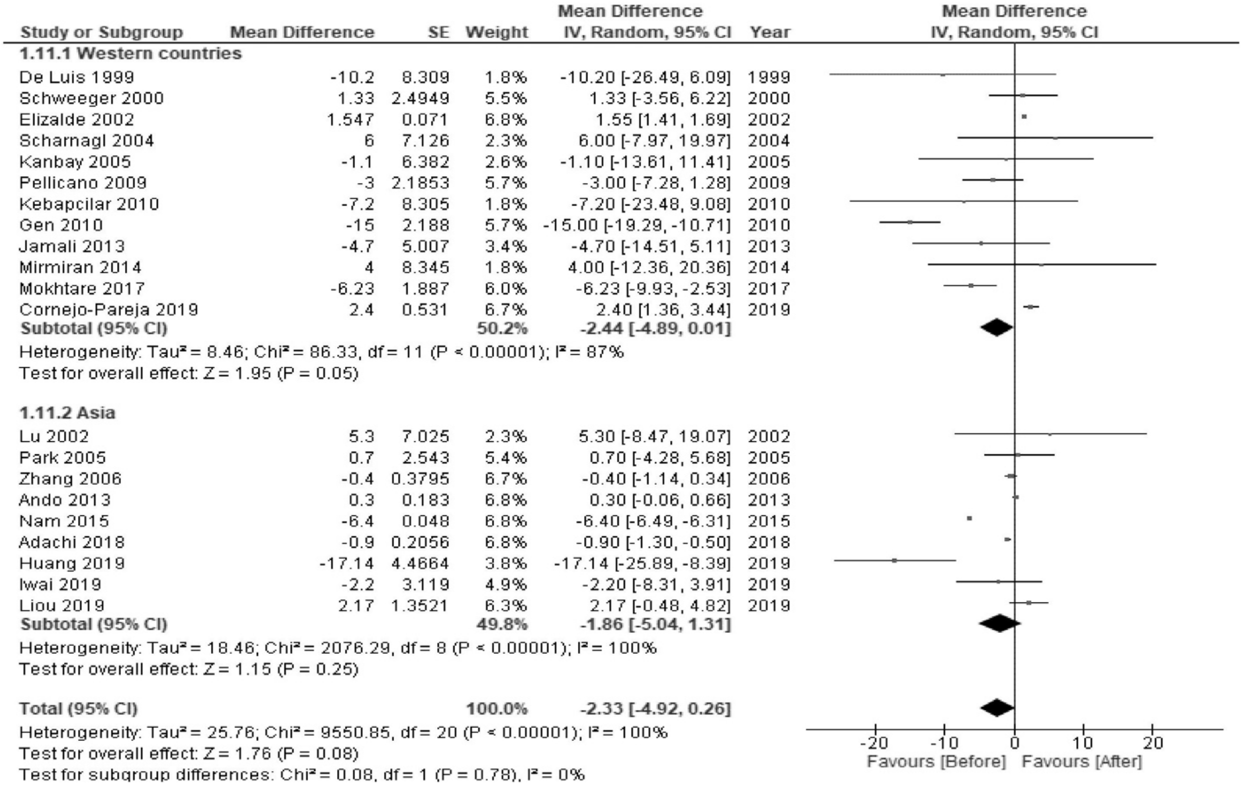

Supplementary Figure S13. Forest plot of the mean changes in low-density lipoprotein cholesterol levels before and after *Helicobacter pylori* eradication in Western countries or Asia

Study or Subgroup	Mean Difference	SE	Weight	Mean Difference IV, Random, 95% CI	Year
**1.12.1 Western countries**					
De Luis 1999	-4.4	10.57	1.9%	-4.40 [-25.12, 16.32]	1999
Schweeger 2000	3.39	2.6888	5.9%	3.39 [-1.88, 8.66]	2000
Elizalde 2002	5.027	0.071	6.8%	5.03 [4.89, 5.17]	2002
Scharnagl 2004	21	7.657	2.9%	21.00 [5.99, 36.01]	2004
Kanbay 2005	-3	6.445	3.5%	-3.00 [-15.63, 9.63]	2005
Kebapcilar 2010	-7.4	9.059	2.4%	-7.40 [-25.16, 10.36]	2010
Gen 2010	-24	4.94	4.4%	-24.00 [-33.68, -14.32]	2010
Jamali 2013	-13.6	5.497	4.1%	-13.60 [-24.37, -2.83]	2013
Mirmiran 2014	8	11.035	1.8%	8.00 [-13.63, 29.63]	2014
Mokhtare 2017	-8.64	2.623	5.9%	-8.64 [-13.78, -3.50]	2017
Cornejo-Pareja 2019	-2.9	1.431	6.5%	-2.90 [-5.70, -0.10]	2019
**Subtotal (95% CI)**			**46.2%**	**-2.97 [-8.44, 2.49]**	
Heterogeneity: Tau² = 56.11; Chi² = 112.54, df = 10 (P < 0.00001); I² = 91%					
Test for overall effect: Z = 1.07 (P = 0.29)					
**1.12.2 Asia**					
Furuta 2002	6.3	0.1972	6.8%	6.30 [5.91, 6.69]	2002
Lu 2002	-0.5	8.632	2.5%	-0.50 [-17.42, 16.42]	2002
Azuma 2002	3.3	20.4929	0.6%	3.30 [-36.87, 43.47]	2002
Kamada 2005	17.1	7.2217	3.1%	17.10 [2.95, 31.25]	2005
Park 2005	4.6	2.916	5.7%	4.60 [-1.12, 10.32]	2005
Zhang 2006	-1.5	0.3305	6.8%	-1.50 [-2.15, -0.85]	2006
Ando 2013	2.4	0.2128	6.8%	2.40 [1.98, 2.82]	2013
Nam 2015	-5.1	0.052	6.8%	-5.10 [-5.20, -5.00]	2015
Liou 2019	1.61	1.5153	6.5%	1.61 [-1.36, 4.58]	2019
Iwai 2019	-0.9	3.507	5.3%	-0.90 [-7.77, 5.97]	2019
Huang 2019	-12.4	8.7394	2.5%	-12.40 [-29.53, 4.73]	2019
**Subtotal (95% CI)**			**53.8%**	**1.29 [-3.15, 5.73]**	
Heterogeneity: Tau² = 39.75; Chi² = 4168.64, df = 10 (P < 0.00001); I² = 100%					
Test for overall effect: Z = 0.57 (P = 0.57)					
**Total (95% CI)**			**100.0%**	**-0.67 [-4.07, 2.72]**	
Heterogeneity: Tau² = 43.89; Chi² = 15108.32, df = 21 (P < 0.00001); I² = 100%					
Test for overall effect: Z = 0.39 (P = 0.70)					
Test for subgroup differences: Chi² = 1.41, df = 1 (P = 0.24), I² = 28.9%					

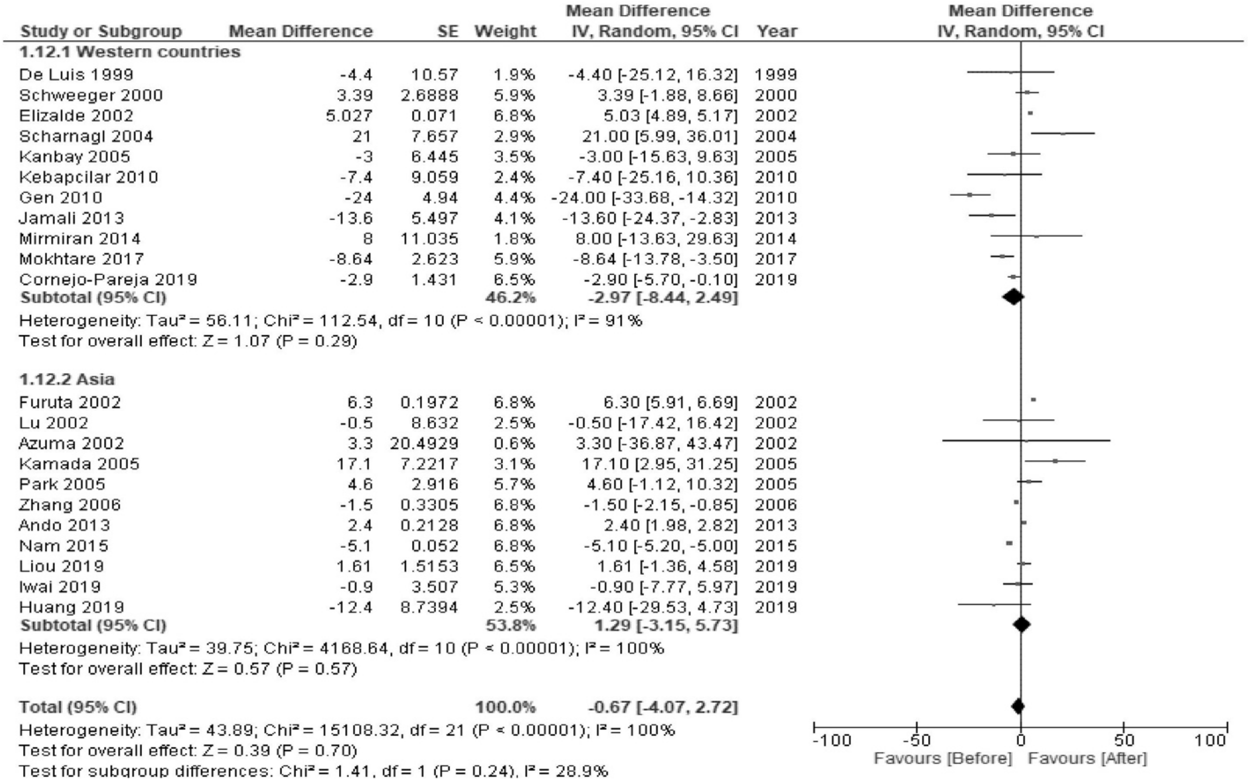

Journal of
Clinical Medicine

MDPI

Supplementary Figure S14. Forest plot of the mean changes in total cholesterol levels before and after *Helicobacter pylori* eradication excluding studies which did not show the levels of HDL-C, TG, LDL-C and TC before *H. pylori* eradication

Study or Subgroup	Mean Difference	SE	Weight	Mean Difference IV, Random, 95% CI	Year
De Luis 1999	5.5	5.266	0.4%	5.50 [-4.82, 15.82]	1999
Schweeger 2000	0	0.7449	8.2%	0.00 [-1.46, 1.46]	2000
Lu 2002	4.2	3.3	1.1%	4.20 [-2.27, 10.67]	2002
Scharnagl 2004	6	1.674	3.3%	6.00 [2.72, 9.28]	2004
Kanbay 2005	2	2.035	2.4%	2.00 [-1.99, 5.99]	2005
Zhang 2006	2.8	0.1659	12.5%	2.80 [2.47, 3.13]	2006
Pellicano 2009	4	0.6899	8.6%	4.00 [2.65, 5.35]	2009
Gen 2010	6	1.962	2.6%	6.00 [2.15, 9.85]	2010
Kebapcilar 2010	-2.2	3.322	1.0%	-2.20 [-8.71, 4.31]	2010
Ando 2013	2.5	0.119	12.7%	2.50 [2.27, 2.73]	2013
Jamali 2013	7.2	0.903	7.0%	7.20 [5.43, 8.97]	2013
Mirmiran 2014	-1.1	1.05	6.0%	-1.10 [-3.16, 0.96]	2014
Adachi 2018	1.3	0.1254	12.7%	1.30 [1.05, 1.55]	2018
Liou 2019	2.53	1.0204	6.2%	2.53 [0.53, 4.53]	2019
Iwai 2019	2.1	1.69	3.3%	2.10 [-1.21, 5.41]	2019
Cornejo-Pareja 2019	2.4	0.531	10.0%	2.40 [1.36, 3.44]	2019
Huang 2019	3.2	2.4003	1.9%	3.20 [-1.50, 7.90]	2019
**Total (95% CI)**			**100.0%**	**2.59 [1.90, 3.28]**	

Heterogeneity: Tau² = 0.97; Chi² = 138.52, df = 16 (P < 0.00001); I² = 88%
Test for overall effect: Z = 7.32 (P < 0.00001)

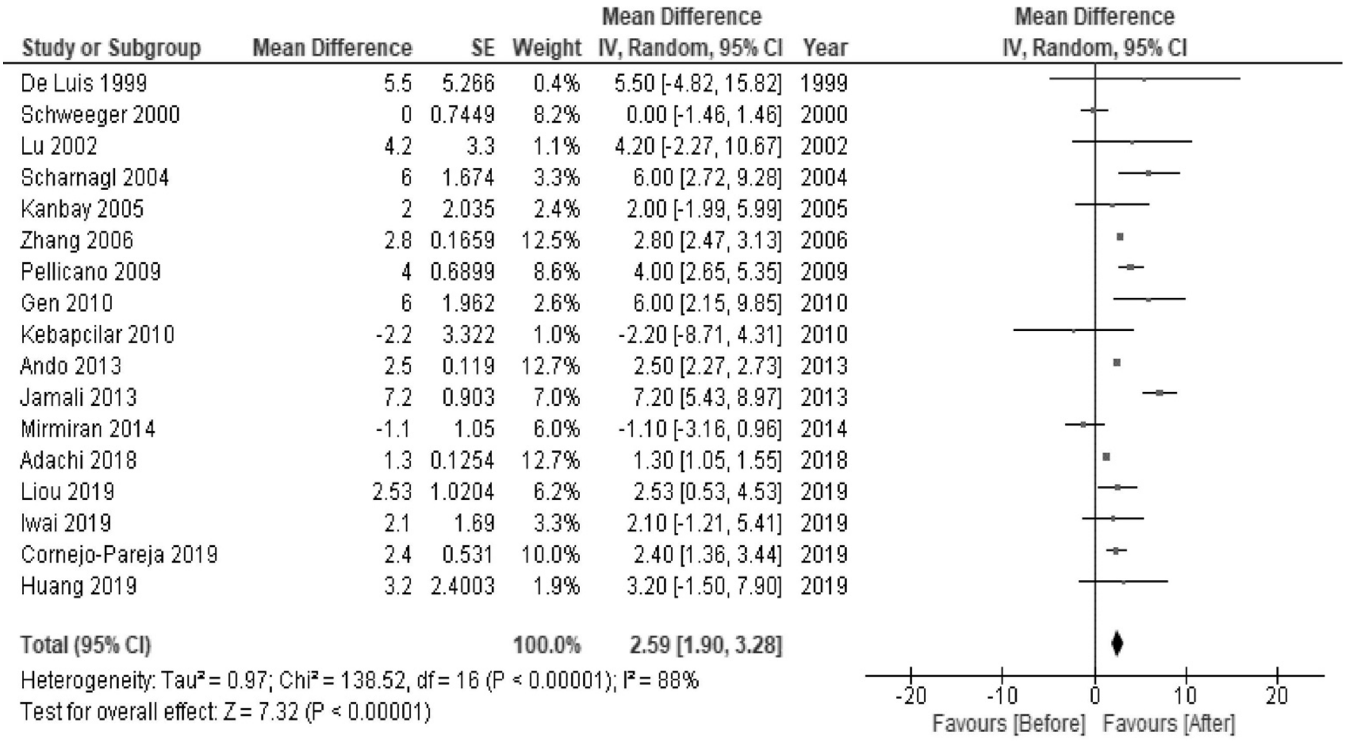

Supplementary Figure S15. Forest plot of the mean changes in high-density lipoprotein cholesterol levels before and after *Helicobacter pylori* eradication excluding studies which did not show the levels of high-density lipoprotein cholesterol before *H. pylori* eradication

Study or Subgroup	Mean Difference	SE	Weight	Mean Difference IV, Random, 95% CI	Year
De Luis 1999	-0.4	18.674	0.4%	-0.40 [-37.00, 36.20]	1999
Schweeger 2000	1.58	5.944	3.4%	1.58 [-10.07, 13.23]	2000
Furuta 2002	6.4	0.6014	17.6%	6.40 [5.22, 7.58]	2002
Lu 2002	-9.6	11.026	1.1%	-9.60 [-31.21, 12.01]	2002
Scharnagl 2004	20	9.885	1.4%	20.00 [0.63, 39.37]	2004
Kanbay 2005	2.5	7.912	2.1%	2.50 [-13.01, 18.01]	2005
Kamada 2005	48.8	27.5796	0.2%	48.80 [-5.26, 102.86]	2005
Pellicano 2009	-3	3.4299	7.4%	-3.00 [-9.72, 3.72]	2009
Gen 2010	-30	13.204	0.8%	-30.00 [-55.88, -4.12]	2010
Kebapcilar 2010	12.4	10.602	1.2%	12.40 [-8.38, 33.18]	2010
Jamali 2013	-54.9	16.434	0.5%	-54.90 [-87.11, -22.69]	2013
Ando 2013	3.3	0.497	17.9%	3.30 [2.33, 4.27]	2013
Mirmiran 2014	23	27.0414	0.2%	23.00 [-30.00, 76.00]	2014
Adachi 2018	1.2	0.8074	17.0%	1.20 [-0.38, 2.78]	2018
Liou 2019	-15.66	4.347	5.4%	-15.66 [-24.18, -7.14]	2019
Iwai 2019	5.4	6.044	3.3%	5.40 [-6.45, 17.25]	2019
Cornejo-Pareja 2019	2.4	0.531	17.8%	2.40 [1.36, 3.44]	2019
Huang 2019	-7.15	7.6429	2.2%	-7.15 [-22.13, 7.83]	2019
**Total (95% CI)**			**100.0%**	**1.33 [-1.04, 3.70]**	

Heterogeneity: Tau² = 7.91; Chi² = 87.84, df = 17 (P < 0.00001); I² = 81%
Test for overall effect: Z = 1.10 (P = 0.27)

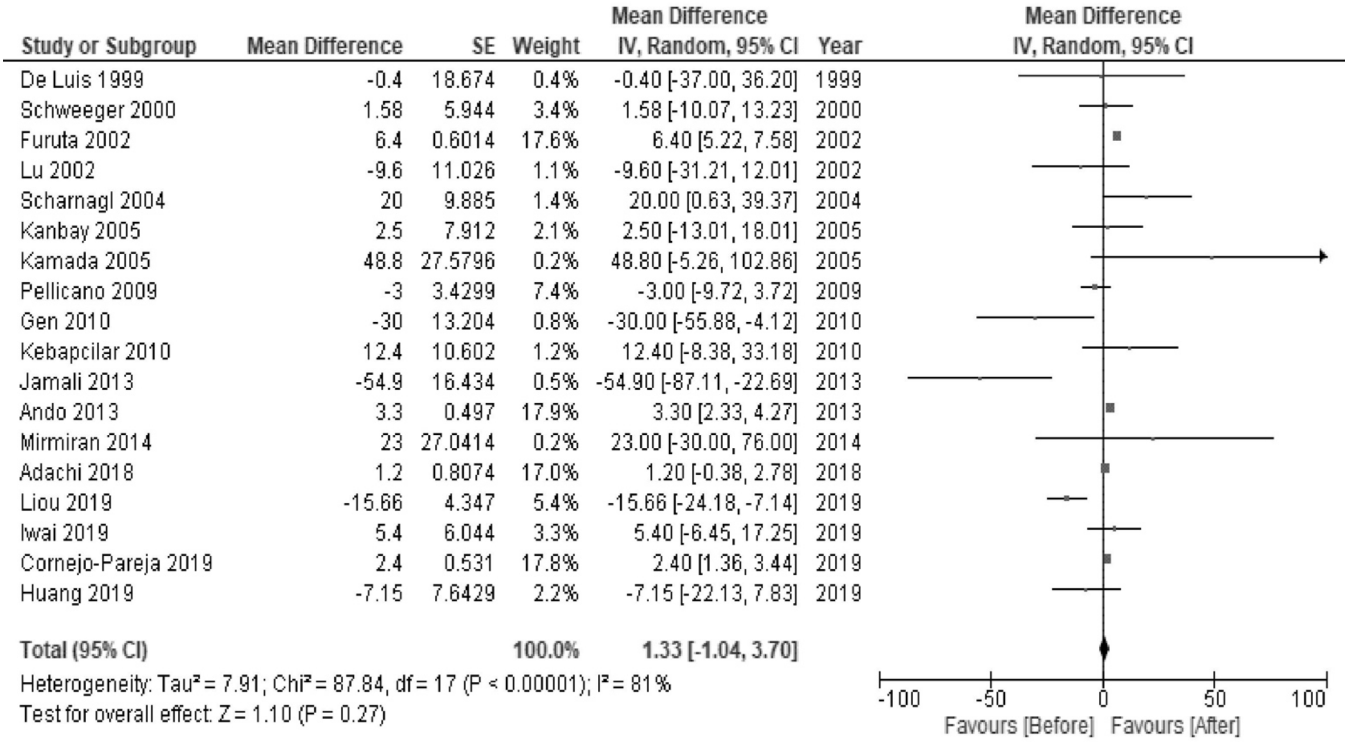

Supplementary Figure S16. Forest plot of the mean changes in triglyceride levels before and after *Helicobacter pylori* eradication excluding studies which did not show the levels of triglycerides before *H. pylori* eradication

Study or Subgroup	Mean Difference	SE	Weight	Mean Difference IV, Random, 95% CI	Year
De Luis 1999	-10.2	8.309	0.5%	-10.20 [-26.49, 6.09]	1999
Schweeger 2000	1.33	2.4949	4.6%	1.33 [-3.56, 6.22]	2000
Lu 2002	5.3	7.025	0.8%	5.30 [-8.47, 19.07]	2002
Scharnagl 2004	6	7.126	0.7%	6.00 [-7.97, 19.97]	2004
Kanbay 2005	-1.1	6.382	0.9%	-1.10 [-13.61, 11.41]	2005
Zhang 2006	-0.4	0.3795	16.0%	-0.40 [-1.14, 0.34]	2006
Pellicano 2009	-3	2.1853	5.5%	-3.00 [-7.28, 1.28]	2009
Kebapcilar 2010	-7.2	8.305	0.5%	-7.20 [-23.48, 9.08]	2010
Gen 2010	-15	2.188	5.5%	-15.00 [-19.29, -10.71]	2010
Jamali 2013	-4.7	5.007	1.4%	-4.70 [-14.51, 5.11]	2013
Ando 2013	0.3	0.183	16.7%	0.30 [-0.06, 0.66]	2013
Mirmiran 2014	4	8.345	0.5%	4.00 [-12.36, 20.36]	2014
Adachi 2018	-0.9	0.2056	16.7%	-0.90 [-1.30, -0.50]	2018
Liou 2019	2.17	1.3521	9.5%	2.17 [-0.48, 4.82]	2019
Iwai 2019	-2.2	3.119	3.2%	-2.20 [-8.31, 3.91]	2019
Cornejo-Pareja 2019	2.4	0.531	15.1%	2.40 [1.36, 3.44]	2019
Huang 2019	-17.14	4.4664	1.8%	-17.14 [-25.89, -8.39]	2019
**Total (95% CI)**			**100.0%**	**-0.97 [-2.19, 0.26]**	

Heterogeneity: Tau² = 2.30; Chi² = 113.43, df = 16 (P < 0.00001); I² = 86%
Test for overall effect: Z = 1.54 (P = 0.12)

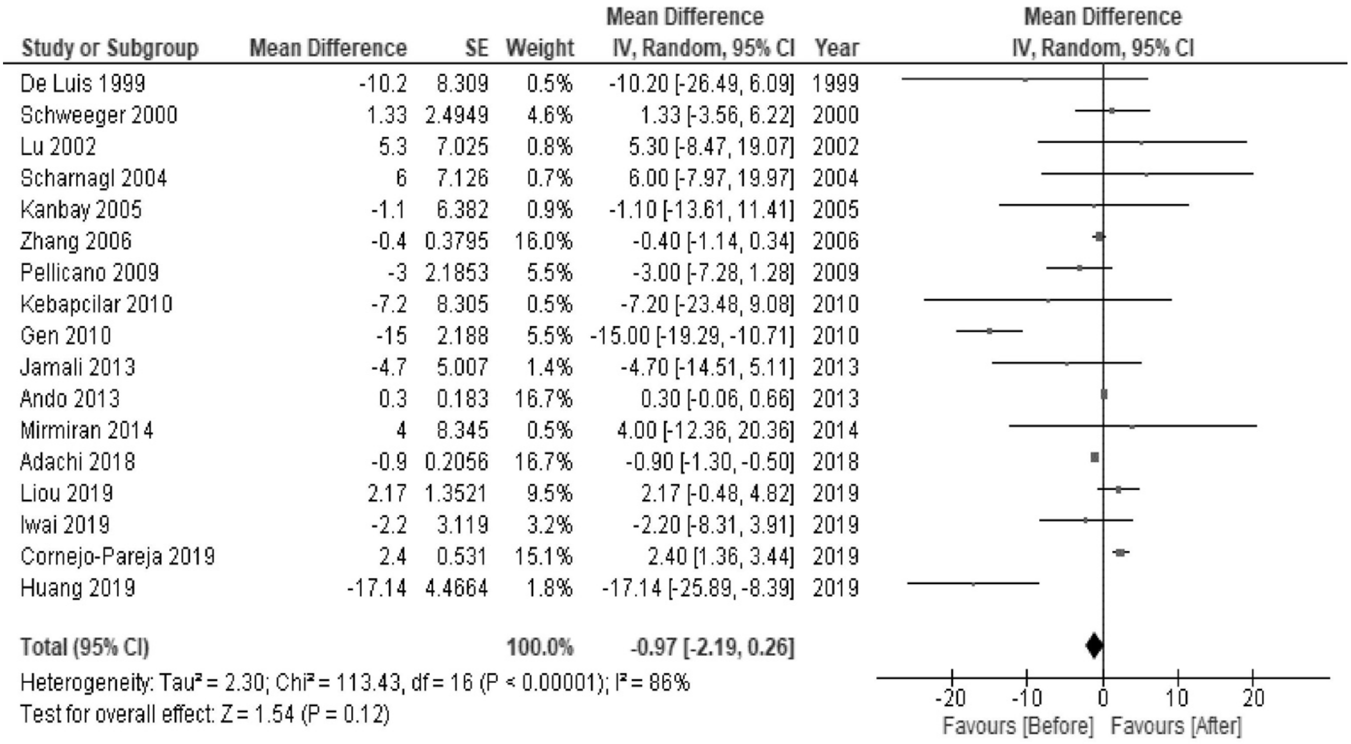

Supplementary Figure S17. Forest plot of the mean changes in low-density lipoprotein cholesterol levels before and after *Helicobacter pylori* eradication excluding studies which did not show the levels of low-density lipoprotein cholesterol before *H. pylori* eradication

Journal of
Clinical Medicine

MDPI

Study or Subgroup	Mean Difference	SE	Weight	Mean Difference IV, Random, 95% CI	Year
De Luis 1999	-4.4	10.5698	1.4%	-4.40 [-25.12, 16.32]	1999
Schweeger 2000	3.39	2.6888	8.3%	3.39 [-1.88, 8.66]	2000
Lu 2002	-0.5	8.6316	2.0%	-0.50 [-17.42, 16.42]	2002
Azuma 2002	3.3	20.4929	0.4%	3.30 [-36.87, 43.47]	2002
Furuta 2002	6.3	0.1972	12.5%	6.30 [5.91, 6.69]	2002
Scharnagl 2004	21	7.657	2.4%	21.00 [5.99, 36.01]	2004
Kanbay 2005	-3	6.445	3.2%	-3.00 [-15.63, 9.63]	2005
Kamada 2005	17.1	7.2217	2.7%	17.10 [2.95, 31.25]	2005
Zhang 2006	-1.5	0.3305	12.5%	-1.50 [-2.15, -0.85]	2006
Kebapcilar 2010	-7.4	9.059	1.8%	-7.40 [-25.16, 10.36]	2010
Gen 2010	-24	4.94	4.6%	-24.00 [-33.68, -14.32]	2010
Ando 2013	2.4	0.2128	12.5%	2.40 [1.98, 2.82]	2013
Jamali 2013	-13.6	5.497	4.0%	-13.60 [-24.37, -2.83]	2013
Mirmiran 2014	8	11.035	1.3%	8.00 [-13.63, 29.63]	2014
Liou 2019	1.61	1.5153	10.8%	1.61 [-1.36, 4.58]	2019
Iwai 2019	-0.9	3.507	6.7%	-0.90 [-7.77, 5.97]	2019
Huang 2019	-12.4	8.7394	1.9%	-12.40 [-29.53, 4.73]	2019
Cornejo-Pareja 2019	-2.9	1.431	11.0%	-2.90 [-5.70, -0.10]	2019
**Total (95% CI)**			**100.0%**	**-0.12 [-2.72, 2.48]**	

Heterogeneity: $Tau^2 = 13.98$; $Chi^2 = 536.20$, df = 17 ($P < 0.00001$); $I^2 = 97\%$
Test for overall effect: Z = 0.09 (P = 0.93)

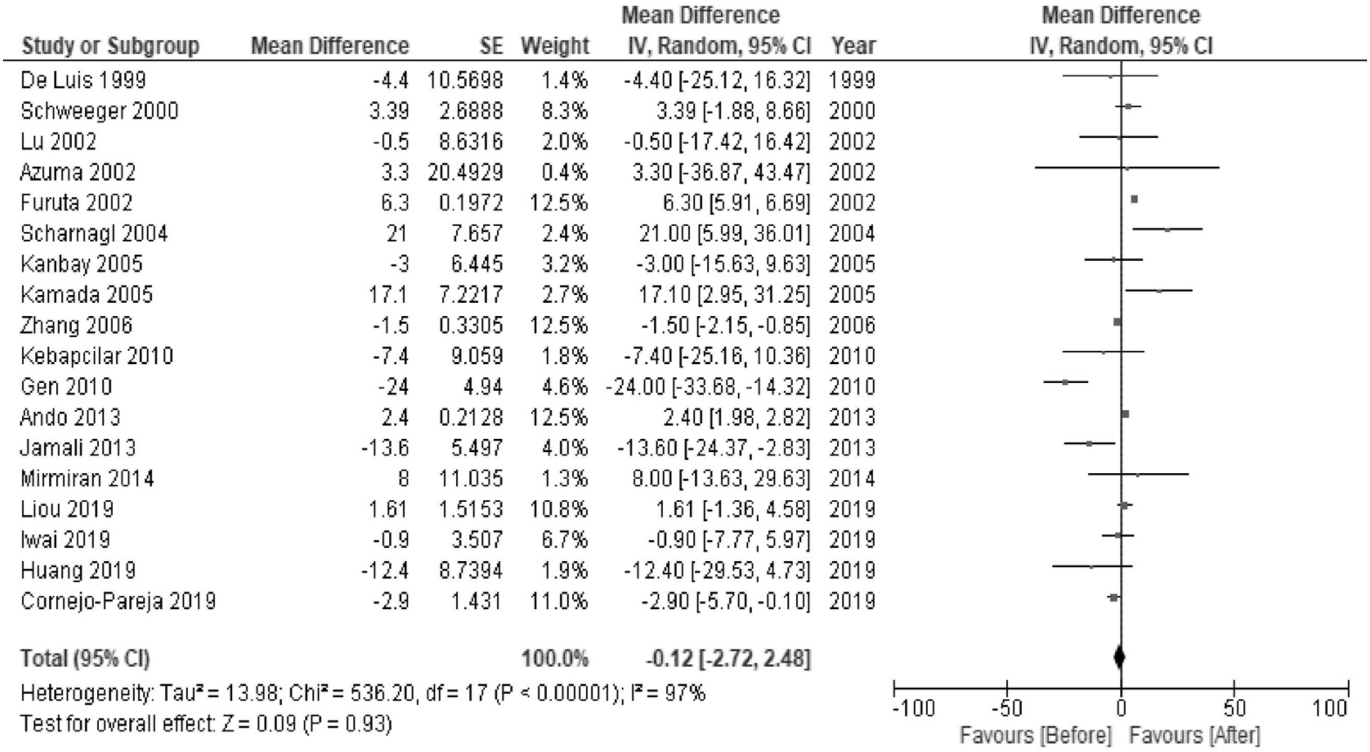

Supplementary Figure S18. Forest plot of the mean changes in total cholesterol levels before and after *Helicobacter pylori* eradication excluding studies which did not show the levels of total cholesterol before *H. pylori* eradication
